# The gut microbiome and mitochondrial function in metabolism, immunity, and disease

**DOI:** 10.1080/19490976.2026.2699451

**Published:** 2026-07-08

**Authors:** Eui Jeong Han, Da-Hye Kim, Jeong Jae Lee, Hea-Jong Chung

**Affiliations:** a Gwangju Center, Korea Basic Science Institute, Gwangju, Republic of Korea; b Department of Convergent Analytical Science, University of Science and Technology, Daejeon, Republic of Korea; c School of Pharmacy, Jeonbuk National University Medical School, Jeonju, Jeonbuk, Republic of Korea

**Keywords:** Gut microbiome, mitochondrial function, immunometabolism, cellular stress response, host–microbe interactions

## Abstract

The gut microbiome is a key regulator of host physiology, yet its effects remain difficult to predict across individuals and contexts. Similar microbial compositions frequently give rise to divergent and delayed phenotypic outcomes, indicating that models based solely on signal strength or steady-state responses are insufficient to explain microbiome-driven host function. In this review, we propose a conceptual perspective in which microbiome-associated variability is shaped by the capacity of host cells to maintain mitochondrial function under persistent metabolic and immune stress. Microbiome-derived metabolites and immune activity define the metabolic and redox environments that constrain mitochondrial performance, thereby influencing how effectively cells recover from repeated stress. When mitochondrial membrane potential, redox balance, and energy production are not fully restored, mitochondria may show increased engagement of quality-control pathways. Over repeated stress–recovery cycles, this pattern may be associated with reduced functional reserve despite preserved baseline activity. This testable perspective may help explain why microbiome-associated phenotypes are delayed, variable, and context-dependent, and it highlights mitochondrial recovery capacity as a potential determinant of disease vulnerability and host–microbiome interactions.

## 1. Introduction

The gut microbiome is widely recognized as a key regulator of host physiology, influencing immunity, metabolism, neural function, and age-associated decline.^1^ ^,^ ^2^ Alterations in microbial composition and metabolite production have been linked to a broad range of diseases, forming the basis of frameworks such as the gut–brain, gut–immune, and gut–metabolic axes.^3^ However, a central challenge remains: similar microbiome configurations can produce markedly different host outcomes, and microbial features alone often fail to predict disease trajectory, resilience, or recovery.^4^ ^,^ ^5^ Microbiome-associated phenotypes are typically delayed, heterogeneous, and systemic, suggesting that current models do not fully explain how microbiome-derived activity is translated into stable physiological states.

Several unresolved gaps limit current explanations of microbiome-associated host phenotypes. First, most microbiome studies still emphasize microbial composition or metabolite abundance, but these variables do not fully explain why comparable microbial states produce different physiological outcomes across individuals, tissues, or disease contexts.^4^ ^,^ ^5^ Second, many microbiome–mitochondria studies rely on steady-state or endpoint readouts, including basal ATP production, basal oxygen consumption, cytokine levels, or metabolite abundance. These measurements can identify altered cellular states, but they do not capture whether mitochondria can restore membrane potential, redox balance, and respiratory reserve after repeated metabolic, inflammatory, or oxidative stress.^4^ ^,^ ^5^ Third, host variables that shape mitochondrial recovery capacity, including genetic background, age, sex or gender, diet, disease status, and baseline immune activity, are often analyzed separately from microbiome-derived metabolic and immune signals.^4^ ^,^ ^5^ As a result, existing models remain limited in explaining why microbiome-associated phenotypes are delayed, heterogeneous, and context-dependent.

This limitation arises in part from the signal-centric logic of prevailing frameworks. Most approaches focus on identifying individual microbial metabolites, cytokines, or endocrine mediators and mapping the pathways they acutely activate.^6^ Implicit in this view is the assumption that host responses scale with signal magnitude. However, microbiome-derived inputs are persistent and recurrent rather than transient. As a result, cellular outcomes are unlikely to be determined solely by signal intensity. Instead, we suggest that microbiome effects are shaped by how repeated exposures influence the ability of cells to recover from stress over time. This shift from signal magnitude to recovery capacity provides a basis for understanding delayed and context-dependent microbiome-associated phenotypes.^7-9^

Mitochondria provide a central system through which these processes can be interpreted. Beyond ATP production, they regulate redox balance, metabolic flexibility, biosynthesis, and cellular tolerance to inflammatory and environmental stress.^10^ ^,^ ^11^ Mitochondrial function is continuously shaped by substrate availability, oxygen tension, cytokine signaling, and intracellular redox conditions. Importantly, mitochondrial performance is defined not only by steady-state activity but also by the ability to restore function following perturbation. This includes recovery of mitochondrial membrane potential (ΔΨm), redox balance (e.g., NAD⁺/NADH), and spare respiratory capacity (SRC).^12^ ^,^ ^13^ When recovery is incomplete or delayed, mitochondria may show increased engagement of quality-control pathways, including PINK1–Parkin–mediated mitophagy.^12^ ^,^ ^13^ In this way, mitochondrial function reflects the cumulative outcome of repeated stress–recovery cycles rather than a static measure of cellular energetics.

The gut microbiome influences these recovery processes by shaping the conditions under which mitochondria operate. Microbiome-derived metabolites, including short-chain fatty acids, tryptophan-derived indoles, and secondary bile acids, regulate substrate availability, membrane stability, and redox balance, thereby defining the environment in which mitochondrial recovery occurs.^14-16^ At the same time, microbiome-dependent immune activity imposes sustained oxidative and energetic pressure through cytokine signaling and altered metabolic demand.^11^ ^,^ ^17^ Rather than acting as isolated triggers of acute damage, these metabolic and immune inputs interact over time to influence whether mitochondrial recovery is complete or progressively impaired.

To address these gaps, we propose a recovery-centered model of microbiome–mitochondria interaction. In this model, microbiome-derived metabolites and microbiome-driven immune activity define the metabolic, redox, and inflammatory conditions under which mitochondria recover after stress. When recovery is efficient, mitochondrial functional reserve is maintained. When recovery is repeatedly delayed or incomplete, mitochondria may show increased engagement of quality-control pathways, which may be associated with reduced mitochondrial recovery capacity despite preserved basal activity. This model provides a testable explanation for the delayed, variable, and context-dependent nature of microbiome-associated phenotypes.

This perspective also has direct implications for experimental design. If microbiome effects are mediated through recovery processes, conventional single-timepoint measurements such as steady-state ATP levels or basal oxygen consumption are insufficient to capture the relevant biology.^18^ Instead, microbiome–host interactions should be investigated using stress–recovery paradigms that quantify parameters such as spare respiratory capacity (SRC), redox recovery following challenge, membrane repolarization kinetics, and mitophagy flux alongside microbial and immune variables.^19^ Such approaches provide a more direct measure of tissue resilience and may help explain the frequent disconnect between microbiome composition and functional outcomes observed in intervention studies.^18-20^

This regulatory logic is particularly relevant in aging. Age-associated decline is characterized by progressive reductions in respiratory capacity, impaired recovery following stress, and increased vulnerability to inflammatory and metabolic challenges, rather than abrupt energetic failure.^21^ ^,^ ^22^ At the same time, aging is associated with reproducible shifts in gut microbiome composition, including reduced abundance of butyrate-producing taxa, altered tryptophan metabolism, and chronic low-grade immune activation. These changes converge on mitochondrial redox balance, substrate utilization, and recovery capacity, suggesting that microbiome-dependent modulation of mitochondrial recovery dynamics contributes to the gradual erosion of cellular resilience observed during aging.^23-25^

In this review, mitochondrial recovery capacity is used to describe how effectively mitochondria return to a stable functional state after metabolic, inflammatory, or oxidative stress. This concept is distinct from basal mitochondrial activity, because cells may maintain resting ATP production while gradually losing the ability to respond to additional stress. Recovery can therefore be evaluated by time-dependent parameters, including the speed and completeness of mitochondrial membrane potential restoration, NAD⁺/NADH rebalance, recovery of ATP-linked respiration, and rebound of spare respiratory capacity after stress.

When these recovery processes are delayed or incomplete, mitochondria may remain in a partially stressed state despite preserved basal activity. Repeated episodes of incomplete recovery can reduce mitochondrial resilience, defined here as the ability to maintain recovery capacity across repeated stress–recovery cycles. This process also narrows functional reserve, meaning the extra respiratory capacity available beyond basal demand. As functional reserve declines, cells operate closer to their bioenergetic limit and become more vulnerable to additional metabolic or inflammatory challenges.

This review is not intended to provide a comprehensive disease-by-disease survey. Instead, it develops a mechanistic perspective in which representative disease contexts are used to illustrate how microbiome-derived metabolic and immune pressures may alter mitochondrial recovery capacity and disease vulnerability.

For experimental interpretability, we use these terms operationally rather than as fixed universal thresholds. Mitochondrial recovery capacity can be quantified as the extent and rate at which stress-altered mitochondrial parameters return toward baseline or matched control levels after a defined perturbation. Recovery failure refers to delayed or incomplete restoration of one or more recovery readouts, such as SRC rebound, ΔΨm repolarization, NAD⁺/NADH restoration, ATP-linked respiration recovery, or mitophagy flux, within a pre-specified recovery window. Mitochondrial resilience refers to the maintenance of recovery capacity across repeated stress–recovery cycles, whereas functional reserve refers to the respiratory capacity available above basal demand, most commonly reflected by SRC. Because these thresholds are likely to vary by cell type, tissue, stressor, and disease context, we do not propose a universal cutoff. Instead, we suggest that future studies predefine recovery windows and report baseline-normalized recovery percentages, recovery half-times or completion times, and cycle-to-cycle changes in recovery efficiency.

## 2. Microbiome–mitochondria signaling and the regulation of mitochondrial recovery

### 2.1. Direct metabolic signaling: microbial metabolites as proximal regulators of mitochondrial function

Microbial metabolites represent the most immediate biochemical interface through which the gut microbiome influences mitochondrial function. Among these, short-chain fatty acids (SCFAs), tryptophan-derived indoles, and secondary bile acids constitute three major classes that regulate mitochondrial behavior through distinct mechanisms that converge on shared recovery-related mitochondrial processes.^26-28^ Rather than acting as isolated signals that directly determine mitochondrial output, these metabolites collectively define the metabolic and redox conditions under which mitochondria attempt to recover following perturbation.

Across tissues, microbiome-derived metabolic inputs influence mitochondrial function through two principal modes. First, they regulate substrate availability and flux into oxidative metabolism, thereby shaping the capacity for energy generation during recovery. Second, they modulate redox balance and inflammatory tone, which constrain the efficiency with which mitochondrial membrane potential, redox equilibrium, and respiratory capacity are restored after stress. Through these combined effects, microbial metabolites do not simply alter mitochondrial activity, but instead establish the boundary conditions that influence the probability that recovery is complete or progressively impaired.

Importantly, these regulatory effects are inherently integrative. Individual metabolites rarely induce overt mitochondrial dysfunction; however, their combined and persistent influence can bias mitochondria toward states of incomplete recovery, increasing the likelihood that stress is carried forward across successive perturbations. In this way, microbiome-derived metabolic signaling contributes to the gradual narrowing of mitochondrial functional reserve and the increased engagement of quality control pathways.

The following sections examine how these regulatory modes emerge across the major metabolite classes and how they converge to shape mitochondrial recovery dynamics.^26^

#### 2.1.1. Short-chain fatty acids: linking microbial fermentation to mitochondrial capacity

Short-chain fatty acids (SCFAs), primarily acetate, propionate, and butyrate, represent the most direct and experimentally tractable interface through which the gut microbiome regulates mitochondrial physiology.^27-29^ Unlike many microbiome–host interactions that operate indirectly through immune or neuroendocrine pathways, SCFAs are chemically defined metabolites whose production, transport, and intracellular metabolism can be quantitatively traced, enabling direct evaluation of their effects on mitochondrial function.^26^ ^,^ ^30^ Importantly, rather than acting simply as metabolic substrates, SCFAs define the biochemical conditions under which mitochondria recover following stress.

In the intestinal epithelium, butyrate serves as a major oxidative substrate for colonocytes and plays a central role in shaping mitochondrial respiration. Microbiota-derived butyrate enters epithelial cells through monocarboxylate transporters (MCT1/SLC16A1) and is converted to acetyl-CoA via mitochondrial *β*-oxidation.^26^ ^,^ ^29^ ^,^ ^31^ This process increases mitochondrial oxygen consumption and lowers epithelial oxygen tension, stabilizing hypoxia-inducible factor (HIF) signaling and promoting transcriptional programs that maintain epithelial barrier integrity.^32^ ^,^ ^33^ Consistent with this mechanism, depletion of butyrate-producing taxa reduces mitochondrial respiration and disrupts barrier-associated gene expression, whereas restoration of luminal butyrate reverses these defects.^34^

The biological relevance of SCFA signaling depends strongly on tissue exposure and concentration gradients. Butyrate reaches high concentrations in the gut lumen and is extensively oxidized by colonocytes, making the intestinal epithelium the primary site of direct butyrate-dependent mitochondrial regulation.^26^ ^,^ ^29^ ^,^ ^31^ As a result, portal and systemic butyrate concentrations are substantially lower than luminal concentrations, and peripheral tissues are unlikely to experience the millimolar butyrate levels often used in vitro. Therefore, mechanisms such as HDAC inhibition, AMPK activation, and PGC-1α-associated mitochondrial remodeling should be interpreted in a tissue- and concentration-dependent manner. In peripheral tissues, SCFA effects may be mediated more by acetate and propionate availability, low-concentration receptor-mediated signaling, immune modulation, endocrine pathways, or long-term metabolic remodeling rather than by direct high-concentration butyrate oxidation.^29^ ^,^ ^35^ ^,^ ^36^

Beyond their role in epithelial metabolism, SCFAs influence mitochondrial function by shaping the capacity for recovery following perturbation. By increasing oxidative flux and supporting efficient coupling of respiration to ATP production, SCFAs enhance the ability of mitochondria to restore membrane potential and redox balance after stress. At the same time, SCFA-driven increases in oxygen consumption reinforce epithelial hypoxia, limiting oxygen diffusion into the gut lumen and stabilizing anaerobic microbial communities, thereby establishing a feedback loop between mitochondrial activity and microbiome structure.^33^ ^,^ ^37^

Outside the intestine, SCFA effects on mitochondrial function should be interpreted more cautiously because tissue exposure differs substantially among acetate, propionate, and butyrate. Acetate and propionate are more systemically available and can contribute to hepatic and peripheral substrate metabolism, whereas butyrate is largely consumed by the colonic epithelium before reaching the systemic circulation.^29^ ^,^ ^35^ ^,^ ^38-41^ Thus, peripheral mitochondrial effects attributed to SCFAs may reflect a combination of systemically available acetate or propionate, receptor-mediated signaling, immune modulation, and indirect metabolic remodeling. Although butyrate can inhibit class I and II histone deacetylases and activate AMPK-related pathways in experimental systems, many of these effects are observed at concentrations higher than those typically achieved in peripheral tissues in vivo.^14^ ^,^ ^35^ ^,^ ^36^ Therefore, peripheral SCFA-dependent mitochondrial recovery should be considered plausible but context-dependent, and should be tested using physiologically relevant concentrations and tissue-specific exposure models.

At the biochemical level, individual SCFAs enter mitochondrial metabolism through complementary routes. Butyrate contributes acetyl-CoA to the tricarboxylic acid cycle, propionate provides anaplerotic input via succinyl-CoA, and acetate expands acetyl-CoA pools that regulate protein acetylation, including modification of mitochondrial enzymes controlling respiratory coupling.^38-41^ These integrated inputs support not only oxidative metabolism but also the restoration of mitochondrial function following perturbation, reinforcing the link between substrate availability and recovery efficiency.

Importantly, SCFA-mediated regulation of mitochondrial function is highly context dependent. In immune cells, SCFAs promote oxidative metabolic states associated with regulatory phenotypes, while under inflammatory conditions they improve coupling efficiency and reduce electron leakage, thereby limiting mitochondrial reactive oxygen species production.^42–45^ These effects do not uniformly increase mitochondrial activity, but instead tune the efficiency and stability of recovery processes across tissues.

These SCFA-dependent effects may contribute to aging-related mitochondrial vulnerability, and their interaction with indole metabolism, bile acid signaling, and immune remodeling is discussed in the integrated aging section below [Table 1].

**Table 1.** Microbial taxa and short-chain fatty acid–mediated regulation of mitochondrial function and recovery-related processes.

Microbial taxa/metabolite context	Major metabolite or activity	Primary host context	Main mechanism	Mitochondrial process affected	Effect on recovery dynamics	Representative references
*Faecalibacterium prausnitzii*	Butyrate	Intestinal epithelium, immune cells	Supports epithelial oxidative metabolism and anti-inflammatory tone	Oxidative phosphorylation, redox buffering, respiratory reserve	Promotes recovery by supporting mitochondrial respiration and limiting inflammatory oxidative stress	Donohoe et al. [26]; Martín et al. [46]
*Roseburia* spp.	Butyrate	Intestinal epithelium, skeletal muscle	Provides butyrate-dependent metabolic support and promotes oxidative phenotype	TCA cycle flux, mitochondrial respiration, metabolic flexibility	Supports sustained recovery by increasing oxidative capacity and substrate availability	Duncan et al. [47]; Den Besten et al. [35]
*Eubacterium rectale*	Butyrate	Intestinal epithelium	Enhances epithelial *β*-oxidation and oxygen consumption	Epithelial mitochondrial respiration, oxygen gradient maintenance	Maintains a recovery-supportive hypoxic epithelial environment	Kelly et al. [32]
*Anaerostipes* spp.	Butyrate from lactate conversion	Colonocytes	Converts lactate to butyrate and supports epithelial energy metabolism	ATP-linked respiration, coupling efficiency	Improves recovery efficiency by supporting mitochondrial coupling and substrate use	Louis and Flint [48]
*Clostridium cluster IV/XIVa*	Butyrate	Gut epithelium, immune cells	Supports regulatory immune tone and anti-inflammatory signaling	Oxidative phosphorylation, inflammatory recovery capacity	Stabilizes recovery under inflammatory stress by reducing immune-driven oxidative burden	Atarashi et al. [49]; Furusawa et al. [42]
*Akkermansia muciniphila*	Acetate; SCFA cross-feeding	Mucosal interface, intestinal epithelium	Supports mucosal metabolism and cross-feeding to butyrate-producing taxa	Indirect support of epithelial respiration and barrier-associated metabolism	Supports recovery indirectly by maintaining a metabolically favorable mucosal environment	Derrien et al. [50]; Everard et al. [51]
*Bacteroides* spp.	Acetate, propionate	Liver, peripheral metabolic tissues	Provides acetyl-CoA and propionate-derived anaplerotic input	TCA cycle anaplerosis, substrate flexibility	Supports recovery by improving metabolic flexibility under variable substrate conditions	Reichardt et al. [52]; Den Besten et al. [35]
*Prevotella* spp.	Propionate	Liver, skeletal muscle	Provides propionate-derived succinyl-CoA input	TCA cycle anaplerosis, substrate adaptation	Enhances recovery capacity by supporting mitochondrial metabolism during substrate fluctuation	Kovatcheva-Datchary et al. [53]
*Ruminococcus bromii*	Acetate; fiber degradation supporting SCFA networks	Gut ecosystem	Degrades resistant starch and supports downstream SCFA production	Indirect support of butyrate-dependent mitochondrial metabolism	Promotes recovery indirectly by maintaining SCFA-producing microbial networks	Ze et al. [54]
*Bifidobacterium* spp.	Acetate; cross-feeding substrate	Intestinal epithelium, immune cells	Produces acetate and supports butyrate-producing taxa through cross-feeding	Redox buffering, epithelial metabolism, SCFA network stability	Reduces recovery failure indirectly by maintaining SCFA-mediated metabolic support	Fukuda et al. [55]

#### 2.1.2. Tryptophan-derived indoles: protective microbiota-derived signals versus toxic host-modified derivatives

Tryptophan-derived indole metabolites represent a chemically diverse group of microbiome-associated signals that can either support or impair mitochondrial recovery depending on metabolite structure, concentration, host modification, receptor engagement, and tissue context. Therefore, indoles should not be interpreted as a uniformly protective metabolite class. Microbiota-derived indoles, including indole-3-propionic acid (IPA), indole-3-aldehyde, and related derivatives, are generally associated with redox stabilization, membrane protection, and maintenance of mitochondrial membrane potential under oxidative or inflammatory stress.^27^ ^,^ ^56-60^ In contrast, host-modified indoles, particularly indoxyl sulfate, can promote oxidative stress, impair respiratory chain activity, and induce mitochondrial membrane depolarization, especially in systemic pathological contexts such as kidney and vascular disease.^61^ ^,^ ^62^ Thus, indole-related effects on mitochondrial recovery should be interpreted according to subclass rather than as a uniform metabolite class: protective microbiota-derived indoles and toxic host-modified derivatives can exert opposing effects on mitochondrial redox balance, membrane potential, and recovery efficiency.

Several microbiota-derived indoles, including indole-3-propionic acid (IPA), indole-3-aldehyde, and related derivatives, attenuate mitochondrial oxidative stress and preserve mitochondrial membrane potential (ΔΨm) under conditions of immune activation or metabolic disturbance.^27^ ^,^ ^57^ Experimental studies consistently demonstrate that IPA reduces mitochondrial reactive oxygen species production, limits lipid peroxidation within mitochondrial membranes, and stabilizes ΔΨm in epithelial, immune, and neuronal cells exposed to oxidative stress.^58-60^ By maintaining membrane polarization, these metabolites may prevent the transition into prolonged depolarized states that impair electron transport chain function and trigger mitochondrial stress signaling, thereby supporting efficient restoration of mitochondrial function following perturbation. However, these protective effects are concentration- and tissue-dependent and should not be generalized to all indole derivatives.

Protective microbiota-derived indole signaling arises through both receptor-independent and receptor-dependent mechanisms. Receptor-dependent effects are mainly mediated by host receptors such as the aryl hydrocarbon receptor (AhR). AhR activation by selected microbiota-derived indoles promotes epithelial barrier maintenance, immune regulation, and antioxidant defense.^27^ ^,^ ^63^ Activation of AhR intersects with NRF2-dependent pathways, increasing expression of enzymes involved in redox buffering and detoxification and thereby reducing oxidative constraints on mitochondrial recovery.^64^ ^,^ ^65^ In contrast, receptor-independent effects include direct scavenging of reactive species, limitation of oxidative damage to mitochondrial lipids and proteins, and preservation of inner mitochondrial membrane integrity.^58-60^ ^,^ ^66^ ^,^ ^67^ These coordinated mechanisms reinforce the ability of mitochondria to restore redox equilibrium following stress.

In mitochondrial terms, AhR/NRF2-associated signaling is relevant to recovery because it can lower the oxidative burden that delays repolarization and respiratory restoration after stress. By increasing antioxidant and detoxification capacity, this pathway may support restoration of ΔΨm, reduce mitochondrial ROS accumulation, preserve respiratory coupling, and limit prolonged mitochondrial stress signaling.^27^ ^,^ ^58-60^ ^,^ ^63-65^ However, these effects should be interpreted as context-dependent recovery-supportive mechanisms rather than uniform consequences of all indole metabolites. This distinction is essential because host-modified indoles such as indoxyl sulfate may produce the opposite mitochondrial outcome by promoting oxidative stress, respiratory impairment, and delayed membrane potential recovery.^61^ ^,^ ^62^

Beyond redox regulation, protective microbiota-derived indoles may contribute to the preservation of mitochondrial membrane architecture. Maintenance of cardiolipin composition within the inner mitochondrial membrane supports electron transport chain organization and limits cytochrome c release during stress. By preventing oxidative modification of cardiolipin and associated lipids, indoles sustain cristae structure and coupling efficiency, both of which are essential for rapid and complete recovery of mitochondrial function following perturbation.^66^ ^,^ ^67^ These membrane-stabilizing effects are particularly important under conditions of repeated inflammatory stress, where cumulative oxidative damage would otherwise impair recovery dynamics.

Host-modified indoles represent a distinct and potentially harmful group. Indoxyl sulfate is generated through host modification of microbiota-derived indole and accumulates particularly under impaired renal clearance or systemic disease conditions.^61^ ^,^ ^62^ Unlike protective microbiota-derived indoles, indoxyl sulfate has been associated with increased oxidative stress, impaired mitochondrial respiration, respiratory chain dysfunction, and mitochondrial membrane depolarization.^61^ ^,^ ^62^ These effects can delay mitochondrial recovery after stress and increase the probability of prolonged redox imbalance, incomplete ΔΨm restoration, and quality-control engagement. Therefore, the biological effect of indole metabolism depends not only on microbial production but also on host conversion, detoxification capacity, clearance, and tissue exposure.

Dose-dependent effects further complicate the interpretation of indole-related signaling because protective microbiota-derived indoles and host-modified indole derivatives can have opposite biological effects. In this review, we use “physiological” and “pathological” exposure in an operational sense rather than as universal concentration cutoffs, because indole exposure differs across the gut lumen, mucosa, circulation, kidney, vascular tissue, and disease state. Physiological or locally balanced exposure to selected microbiota-derived indoles, such as IPA, IAld, and ILA, may support epithelial barrier function, redox balance, mitochondrial membrane stability, and recovery efficiency.^27^ ^,^ ^56-60^ ^,^ ^64^ ^,^ ^65^ In contrast, pathological exposure refers to excessive indole availability, altered host conversion, or impaired clearance that shifts the indole pool toward toxic host-modified derivatives, particularly indoxyl sulfate.^61^ ^,^ ^62^ ^,^ ^68^ Under these conditions, indole-related metabolism may increase oxidative burden, impair respiratory activity, delay ΔΨm restoration, and reduce mitochondrial recovery efficiency.^61^ ^,^ ^62^ Thus, the mitochondrial consequence of indole metabolism depends on the composition of the indole pool, tissue exposure, host modification and clearance capacity, and whether receptor-mediated protective signaling or toxic systemic accumulation predominates.^27^ ^,^ ^56-65^ ^,^ ^68-71^

Tissue specificity is also critical. In intestinal epithelial cells, protective microbiota-derived indoles may help maintain barrier integrity, redox balance, and mitochondrial membrane stability.^27^ ^,^ ^57^ In immune cells, they may reduce inflammatory amplification and oxidative burden through AhR- and NRF2-associated signaling.^27^ ^,^ ^64^ ^,^ ^65^ In neuronal cells, IPA has been linked to protection against oxidative injury and preservation of mitochondrial function.^58-60^ In contrast, in kidney and vascular tissues, accumulation of toxic host-modified indoles such as indoxyl sulfate may promote oxidative stress, respiratory impairment, and delayed mitochondrial recovery.^61^ ^,^ ^62^ These tissue-dependent effects help explain why protective microbiota-derived indoles can support mitochondrial recovery in some contexts, whereas host-modified derivatives such as indoxyl sulfate can impair recovery in others.

Functionally, indoles have a dual role in mitochondrial recovery. Microbiota-derived protective indoles can support recovery by reducing mitochondrial ROS, preserving ΔΨm, maintaining membrane integrity, and activating antioxidant pathways.^27^ ^,^ ^56-60^ ^,^ ^64^ ^,^ ^65^ In contrast, host-modified or excessive indoles can impair recovery by increasing oxidative stress, disrupting ΔΨm, inhibiting respiratory activity, and prolonging inflammatory or metabolic stress.^61^ ^,^ ^62^ Therefore, mitochondrial recovery is not determined by indole production alone, but by the composition of the indole pool, the balance between protective and toxic derivatives, the degree of host modification, and the tissue environment.

Crucially, these effects extend beyond individual stress events and accumulate over time. When mitochondrial recovery remains efficient, functional capacity is maintained across repeated challenges. In contrast, even modest impairments in recovery can compound across cycles, progressively reducing mitochondrial reserve and increasing susceptibility to subsequent stress. In this way, recovery dynamics provide a mechanistic link through which persistent microbiome-derived signals are translated into durable cellular states.

Age-related shifts in tryptophan metabolism may contribute to mitochondrial vulnerability by reducing protective microbiota-derived indoles and/or increasing harmful host-modified indole derivatives; these effects are integrated with SCFA availability, bile acid signaling, inflammation, and immunosenescence in the aging section below [Table 2].

**Table 2.** Context-dependent effects of microbiota-derived and host-modified indoles on mitochondrial recovery.

Microbial taxa/metabolite context	Major metabolite or activity	Primary host context	Main mechanism	Mitochondrial process affected	Effect on recovery dynamics	Representative references
*Clostridium sporogenes*	Indole-3-propionic acid (IPA)	Intestinal epithelium, brain	Produces IPA, a microbiota-derived antioxidant indole	Mitochondrial ROS, ΔΨm, lipid peroxidation	Supports recovery by limiting oxidative damage and preserving membrane potential	Wikoff et al. [72]; Dodd et al. [56]
*Peptostreptococcus anaerobius*	IPA	Gut epithelium	Contributes to IPA production and membrane-protective indole signaling	Lipid peroxidation, mitochondrial membrane integrity	Enhances recovery efficiency under oxidative stress by maintaining membrane stability	Dodd et al. [56]
*Lactobacillus* spp.	Indole-3-aldehyde (IAld), indole derivatives	Intestinal epithelium, immune cells	Activates AhR-associated epithelial and immune regulatory pathways	Redox buffering, antioxidant defense, inflammatory tone	Promotes recovery by reducing inflammatory amplification and supporting antioxidant programs	Zelante et al. [27]
*Bifidobacterium* spp.	Indole-3-lactic acid (ILA)	Intestinal epithelium, immune cells	Supports antioxidant and epithelial-protective signaling	Mitochondrial ROS, redox balance	Supports recovery by enhancing redox buffering and limiting oxidative stress	Henrick et al. [73]
*Akkermansia muciniphila*	Indole-related metabolites indirect	Mucosal interface, intestinal epithelium	Maintains mucosal metabolic environment and barrier-associated signaling	Indirect support of ΔΨm stability and epithelial mitochondrial function	Supports recovery indirectly by preserving mucosal homeostasis and reducing epithelial stress exposure	Derrien et al. [50]; Shin et al. [74]
*Escherichia coli*	Indole	Gut lumen, epithelium	Produces indole with dose- and context-dependent redox effects	Redox balance, mitochondrial stress signaling	Low or physiological exposure may support epithelial homeostasis, whereas excessive exposure may impair recovery	Lee et al. [69]; Hirakawa et al. [70]
*Proteus* spp.	Indole	Gut lumen, dysbiotic context	Excess indole production under dysbiosis-associated conditions	Oxidative burden, redox imbalance	Promotes recovery failure by increasing oxidative stress and destabilizing redox balance	Roager and Licht [71]
*Enterococcus* spp./systemic host modification	Host-modified indole derivatives	Systemic pathological contexts	Associated with generation or accumulation of host-modified indole products	Mitochondrial ROS, respiratory efficiency	Impairs recovery by increasing oxidative stress and reducing respiratory efficiency	Barreto et al. [68]
Uremia-associated host–microbe metabolism	Indoxyl sulfate	Kidney, vascular tissue	Host-modified toxic indole accumulating under impaired clearance	ROS, ΔΨm, ETC activity	Strongly promotes recovery failure by inducing mitochondrial toxicity, depolarization, and respiratory impairment	Dou et al. [61]
Protective microbiota-derived indole pool	IPA, IAld, ILA	Intestinal epithelium, immune cells, neuronal cells	AhR/NRF2-associated signaling and receptor-independent antioxidant activity	Redox balance, membrane integrity, ΔΨm	Supports recovery by stabilizing redox conditions and preserving mitochondrial membrane function	Zelante et al. [27]; Dodd et al. [56]; Venkatesh et al. [57]; Chyan et al. [58]; Poeggeler et al. [59]; Geddo et al. [60]; Grishanova and Perepechaeva [64]; Dietrich [65]
Toxic host-modified indole pool	Indoxyl sulfate and related derivatives	Kidney, vascular tissue, systemic disease states	Impaired clearance, oxidative stress, direct mitochondrial injury	ROS, ΔΨm loss, ETC inhibition	Promotes recovery failure by prolonging oxidative stress and impairing membrane potential restoration	Dou et al. [61]; Ellis et al. [62]; Barreto et al. [68]
Dose- and tissue-dependent indole effects	Indole derivatives depending on concentration and tissue exposure	Gut epithelium, immune cells, brain, kidney, vascular tissue	Concentration-, host modification-, and tissue-specific signaling	Redox buffering, inflammatory tone, mitochondrial membrane stability	Explains why indoles may support recovery in some contexts but impair recovery under dysbiosis or systemic disease	Zelante et al. [27]; Venkatesh et al. [57]; Dou et al. [61]; Ellis et al. [62]; Lee and Lee [69]; Hirakawa et al. [70]; Roager and Licht [71]; Barreto et al.[68]

#### 2.1.3. Microbiota-modified bile acids: receptor signaling, mitochondrial toxicity, and emerging molecular diversity

Microbiota-modified bile acids represent a broad signaling and metabolic interface between the gut microbiome and host mitochondria. In the previous classical view, microbial bile acid metabolism was mainly described through deconjugation and 7α-dehydroxylation of primary bile acids, generating conventional secondary bile acids such as deoxycholic acid (DCA) and lithocholic acid (LCA).^16^ ^,^ ^28^ ^,^ ^75^ However, recent studies have expanded this view by showing that gut microbes generate a much broader bile acid pool, including amino acid-conjugated bile acids, bacterial bile acid amidates, isomeric and epimeric bile acids, acylated or dicarboxylic acid-conjugated bile acids, and structurally modified LCA derivatives. These discoveries indicate that microbiota-dependent bile acid signaling cannot be fully explained by DCA and LCA alone.^76-80^

At physiological or moderate concentrations, secondary bile acids regulate mitochondrial metabolism mainly through receptor-dependent pathways. TGR5 activation can increase cAMP signaling, promote energy expenditure, and enhance oxidative metabolism in metabolically active tissues, whereas FXR regulates bile acid synthesis, lipid metabolism, glucose homeostasis, and mitochondrial substrate handling.^16^ ^,^ ^28^ ^,^ ^75^ ^,^ ^81-84^ However, the transition from receptor-mediated signaling to mitochondrial toxicity is not defined by a single universal threshold. Instead, it depends on bile acid hydrophobicity, concentration, conjugation state, tissue exposure, transporter activity, and host detoxification capacity. When hydrophobic bile acids accumulate beyond tissue-specific buffering capacity, they may directly disrupt mitochondrial membrane integrity, increase membrane permeability, elevate mitochondrial ROS, impair electron transport chain activity, and reduce ATP production.^85^ ^,^ ^86^

We therefore frame bile acid effects on mitochondrial recovery using a functional threshold model. In this model, bile acids may support recovery-related metabolism when receptor-mediated signaling through pathways such as TGR5 and FXR predominates within a tissue’s buffering and detoxification capacity.^16^ ^,^ ^28^ ^,^ ^75^ ^,^ ^81-84^ However, when hydrophobic bile acids such as LCA or DCA exceed tissue-specific handling capacity, the dominant effect may shift from receptor-mediated adaptation to receptor-independent mitochondrial stress. This shift may involve mitochondrial membrane destabilization, increased ROS generation, impaired electron transport, reduced ATP production, delayed ΔΨm restoration, and reduced respiratory recovery.^85^ ^,^ ^86^ Thus, the relevant threshold is not a single universal concentration, but a context-dependent boundary determined by hydrophobicity, receptor potency, conjugation state, transporter activity, tissue exposure, and host detoxification capacity. This threshold may be crossed under different disease-related conditions, including cholestatic liver disease, colorectal cancer-associated or high-risk intestinal environments, and post-antibiotic dysbiosis, in which microbial bile acid transformation and host clearance capacity are altered.^87-89^

Bile acid potency also differs substantially among individual molecules. LCA is generally more potent than DCA in TGR5 activation and is among the strongest endogenous TGR5 agonizts.^16^ ^,^ ^90^ This higher potency is biologically relevant because LCA is also highly hydrophobic and can directly affect mitochondrial membrane stability when present at excessive concentrations. In liver mitochondria, LCA has been shown to induce Ca^2^⁺-dependent permeability changes, mitochondrial swelling, and collapse of mitochondrial membrane potential.^91^ Therefore, LCA and DCA should not be treated as equivalent secondary bile acids. Rather, their mitochondrial effects depend on receptor potency, hydrophobicity, concentration, and tissue-specific exposure. This distinction is important for the recovery framework because LCA may exert strong receptor-mediated metabolic signaling at lower exposure levels, whereas excessive LCA exposure may more readily cross the functional threshold into membrane-associated mitochondrial stress.^16^ ^,^ ^90^ ^,^ ^91^ DCA may also contribute to mitochondrial stress under pathological exposure, but its receptor potency and toxicity profile should not be assumed to be identical to those of LCA.^16^ ^,^ ^85^ ^,^ ^86^ ^,^ ^90^ ^,^ ^91^

Tissue context further influences whether bile acids are associated with recovery-supportive signaling or mitochondrial stress. In the intestine, bile acids influence epithelial barrier function, enteroendocrine signaling, immune tone, and microbial community structure.^28^ ^,^ ^92^ ^,^ ^93^ In the liver, FXR-dependent signaling regulates bile acid synthesis and lipid metabolism, while excessive hydrophobic bile acids can impose mitochondrial stress through membrane disruption and oxidative injury.^83-86^ In skeletal muscle and adipose tissue, TGR5 activation is linked to increased energy expenditure, fatty acid oxidation, thermogenic signaling, and metabolic flexibility.^16^ ^,^ ^81^ ^,^ ^82^ Thus, the same bile acid may have different mitochondrial consequences depending on whether the dominant context is intestinal signaling, hepatic detoxification, muscle oxidative metabolism, or adipose thermogenesis.

Recent discoveries of microbiota-modified bile acids further complicate this signaling landscape. Bacterial bile salt hydrolases, classically known for deconjugating host bile acids, can also act as amine *N*-acyltransferases that generate bacterial bile acid amidates.^76^ These bacterial bile acid amidates can activate host ligand-responsive receptors, including PXR and AhR, thereby expanding the receptor space beyond classical FXR and TGR5 signaling.^76^ In addition, longitudinal human studies have identified diverse microbially conjugated bile acids and linked altered ursodeoxycholic acid and deoxycholic acid conjugates to immune development and islet autoimmunity.^77^ Other metabolomic studies have detected large numbers of amino acid-conjugated bile acids, including isomeric and epimeric forms, in human and mouse fecal samples.^78^ More recently, tryptophan-conjugated cholic acid was identified as a microbial amino acid-conjugated bile acid that improves glucose homeostasis through the orphan receptor MRGPRE rather than through classical FXR or TGR5 signaling.^79^ These findings show that microbiota-derived bile acid biology now includes receptor activities and host effects that extend beyond conventional secondary bile acids. Mechanistically, these newly identified bile acid classes are relevant to mitochondrial recovery not because they have all been shown to directly regulate mitochondria, but because they expand the receptor, immune, endocrine, and metabolic pathways that define the mitochondrial stress environment.^76-80^ For example, bacterial bile acid amidates can engage ligand-responsive receptors such as PXR and AhR, whereas amino acid-conjugated bile acids and related derivatives may influence metabolic and enteroendocrine signaling through receptor pathways beyond classical FXR and TGR5.^76^ ^,^ ^78^ ^,^ ^79^ Therefore, their contribution to recovery dynamics should currently be interpreted mainly as indirect and context-dependent, pending direct validation of their effects on ΔΨm recovery, SRC rebound, mitochondrial ROS, and mitophagy flux.^76-80^

Functionally, microbiota-modified bile acids should therefore be viewed as context-dependent regulators of mitochondrial recovery. Classical DCA and LCA can support metabolic adaptation through TGR5 and FXR signaling within physiological ranges but may impair recovery when hydrophobic bile acids accumulate and directly damage mitochondrial membranes. Newly identified bile acid derivatives may further regulate mitochondrial recovery indirectly by modulating immune tone, glucose metabolism, lipid handling, enteroendocrine signaling, and receptor pathways beyond FXR and TGR5. Accordingly, the net effect of bile acid metabolism on mitochondrial recovery depends on molecular structure, potency, receptor selectivity, concentration, tissue exposure, and the balance between signaling and toxicity [Table 3].

**Table 3.** Microbial taxa, classical secondary bile acids, and newly identified microbiota-modified bile acids regulating mitochondrial recovery.

Microbial taxa/metabolite context	Major metabolite or activity	Primary host context	Main mechanism	Mitochondrial process affected	Effect on recovery dynamics	Representative references
*Clostridium* spp. *Clusters XIVa, XI*	7α-dehydroxylation	DCA, LCA	TGR5, FXR	Oxidative metabolism and substrate balance	Defines baseline recovery conditions by shaping secondary bile acid composition	Ridlon JM et al. [94]; Buffie CG et al. [87]
*Clostridium scindens*	Primary-to-secondary bile acid conversion	DCA, LCA	TGR5	Fatty acid oxidation and energy demand during recovery	Enhances recovery-associated oxidative capacity under physiological conditions	Studer N et al. [88]
*Eubacterium* spp.	Bile acid modification	DCA	FXR indirect	Mitochondrial substrate availability	Alters hepatic metabolic context that determines recovery efficiency	Gérard P [95]
*Bacteroides* spp.	Bile salt hydrolase activity	Deconjugated bile acids	FXR, PXR, AhR indirect/context-dependent	Substrate delivery, bile acid pool composition, inflammatory tone	Shapes recovery conditions by controlling bile acid conjugation state and receptor signaling	Joyce SA et al. [96]; Rimal B et al. [76]
*Lactobacillus* spp.	Bile salt hydrolase activity	Deconjugated bile acids; bacterial bile acid amidates	FXR, PXR, AhR context-dependent	Bile acid-induced mitochondrial stress and receptor-active bile acid pools	Preserves recovery efficiency under physiological conditions by limiting hydrophobic bile acid toxicity	Jones ML et al. [97]; Rimal B et al. [76]
*Bifidobacteriu*m spp.	Bile salt hydrolase activity	Deconjugated bile acids	FXR indirect; PXR/AhR context-dependent	Mitochondrial membrane stability indirect	Supports stable recovery conditions by contributing to balanced bile acid composition	Tanaka H et al. [98]; Rimal B et al. [76]
Dysbiosis-associated Clostridia	Excess bile acid conversion	DCA, LCA at high levels	Receptor-independent at toxic exposure levels	Membrane permeability, oxidative stress, ΔΨm stability	Promotes recovery failure through ROS accumulation, membrane disruption, and impaired ΔΨm restoration	Bernstein H et al. [89]; Krähenbühl S et al. [86]
Pathological bile acid accumulation	Host–microbe imbalance	DCA, LCA; hydrophobic bile acid accumulation	None/receptor-independent	ETC dysfunction, membrane destabilization, reduced ATP production	Impairs recovery capacity when tissue-specific buffering, transport, and detoxification capacity are exceeded	Krähenbühl S et al. [86]
LCA-dominant hydrophobic bile acid exposure	Potency-differentiated secondary bile acid signaling	LCA>DCA	TGR5; receptor-independent toxicity at high exposure	Strong receptor activation at physiological levels; mitochondrial membrane stress at excessive levels	Distinguishes LCA from DCA because LCA has stronger TGR5 potency and greater membrane-associated mitochondrial stress potential	Duboc H et al. [90]; Watanabe M et al. [16]
Balanced microbial consortium	Homeostatic bile acid cycling	Mixed secondary bile acids	TGR5+FXR	Metabolic flexibility and mitochondrial efficiency	Maintains recovery efficiency across repeated stress cycles within a physiological signaling window	Wahlström A et al. [28]
Amino acid-conjugated bile acid pool	Microbial amino acid conjugation	PheCA, TyrCA, LeuCA, Trp-CA and related MABAs	FXR, PXR, AhR, MRGPRE	Metabolic, immune, and enteroendocrine signaling	Expands bile acid signaling beyond DCA/LCA and may indirectly influence mitochondrial recovery through systemic metabolic and immune pathways	Quinn RA et al. [80]; Wang YZ et al. [78]; Lin J et al. [79]
Bacterial bile acid amidates	BSH-dependent amine *N*-acyltransferase activity	BBAAs; amine-conjugated bile acids	PXR, AhR	Host ligand-responsive transcriptional pathways	May indirectly affect recovery by regulating immune tone, detoxification pathways, and metabolic signaling	Rimal B et al. [76]
Isomeric and epimeric amino acid-conjugated bile acids	Structure-specific microbial/host bile acid modification	AA-BA isomers and epimers	Structure-dependent receptor activity	Fine-tuning of receptor signaling and metabolic response	Shows that bile acid effects depend on molecular identity, stereochemistry, and conjugation pattern rather than total abundance alone	Wang YZ et al. [78]
High-activity metabolic bile acids	Microbial amino acid-conjugated bile acid signaling	Trp-CA	MRGPRE; GLP-1-related metabolic signaling	Glucose metabolism and systemic metabolic homeostasis	May support recovery indirectly by improving metabolic homeostasis and reducing substrate stress	Lin J et al. [79]
Acylated or carbon skeleton-modified bile acid derivatives	Expanded microbial bile acid chemistry	Acylated bile acids; carbon skeleton-modified bile acids; structurally modified LCA derivatives	Context-dependent; incompletely defined	Immune and metabolic pathways that shape mitochondrial stress environments	May alter recovery dynamics indirectly through receptor selectivity, immune tone, and metabolic remodeling; direct mitochondrial effects require further validation	Rimal B et al. [76]; Wang YZ et al. [78]

#### 2.1.4. Convergent mitochondrial features shaped by microbial metabolites

Although short-chain fatty acids, tryptophan-derived indoles, and secondary bile acids act through distinct molecular pathways, their effects converge on a limited set of mitochondrial features that influence the probability that recovery following stress is successful or progressively impaired. However, convergence at the level of mitochondrial outcomes should not be interpreted as mechanistic equivalence among these metabolite classes. Across tissues, these microbial metabolites repeatedly influence mitochondrial oxidative capacity, redox balance, membrane integrity, and susceptibility to stress-induced turnover. Rather than uniformly increasing or suppressing mitochondrial activity, microbial metabolites define the conditions under which mitochondria are able or unable to restore functional homeostasis.^99-101^

The three metabolite classes differ in their primary modes of action. Short-chain fatty acids primarily act as metabolic substrates and epigenetic regulators. They support mitochondrial recovery by providing carbon sources for oxidative metabolism, promoting tricarboxylic acid cycle flux, improving coupling of respiration to ATP production, and regulating metabolic gene expression through mechanisms such as histone deacetylase inhibition and AMPK-related signaling. In contrast, tryptophan-derived indoles stabilize redox conditions and membrane integrity, limiting oxidative damage and preserving membrane potential during inflammatory or metabolic stress. Their effects are mediated through receptor-dependent pathways, including aryl hydrocarbon receptor and NRF2-associated antioxidant signaling, as well as receptor-independent mechanisms such as direct antioxidant activity and mitochondrial lipid protection. Secondary bile acids regulate substrate balance and metabolic efficiency through receptor-dependent signaling while introducing a concentration-dependent threshold beyond which membrane integrity and respiratory function deteriorate. Unlike SCFAs and indoles, bile acids act mainly as systemic signaling molecules through TGR5 and FXR, but excessive accumulation of hydrophobic bile acids can shift their action from physiological signaling to direct mitochondrial toxicity.^25^ ^,^ ^27^ ^,^ ^102^ Together, these metabolite classes do not act redundantly but instead define distinct constraints on mitochondrial function that collectively shape mitochondrial recovery capacity.

A concise comparison of these metabolite classes clarifies their convergence and divergence. SCFAs primarily influence mitochondrial recovery by shaping substrate availability, TCA cycle input, respiratory coupling, and epigenetic or AMPK-associated metabolic regulation.^26^ ^,^ ^29^ ^,^ ^35^ ^,^ ^36^ ^,^ ^38-45^ Microbiota-derived protective indoles mainly influence recovery by stabilizing redox balance, preserving ΔΨm, protecting mitochondrial membrane integrity, and engaging AhR/NRF2-associated antioxidant pathways, whereas host-modified indoles such as indoxyl sulfate may impair recovery through oxidative stress, respiratory dysfunction, and membrane depolarization.^27^ ^,^ ^56-65^ Bile acids primarily influence recovery through receptor-mediated systemic signaling via TGR5, FXR, PXR, AhR, and related pathways, but hydrophobic bile acids such as LCA and DCA may impair recovery when tissue-specific buffering and detoxification capacity are exceeded.^16^ ^,^ ^28^ ^,^ ^75-86^ ^,^ ^90^ ^,^ ^92^ ^,^ ^93^ Thus, these metabolites converge on common recovery-related outcomes, including ΔΨm restoration, SRC rebound, redox recovery, respiratory efficiency, and mitophagy engagement, but they reach these outcomes through distinct and non-redundant mechanisms.^18-20^ ^,^ ^99-105^

This distinction is important because similar downstream mitochondrial outcomes, such as reduced SRC, delayed ΔΨm restoration, redox imbalance, or increased mitochondrial stress signaling, may arise from different upstream microbial metabolite disturbances. Therefore, metabolite class, tissue exposure, and disease context should be considered when interpreting microbiome-associated mitochondrial recovery phenotypes.^18-20^ ^,^ ^99-101^

The convergence of microbial metabolite signaling becomes most apparent in the context of repeated stress–recovery cycles. By jointly shaping oxidative capacity, redox buffering, and membrane stability, microbial metabolites influence how efficiently mitochondria restore membrane potential, redox balance, and ATP production following transient perturbations. When metabolite composition remains balanced, recovery is rapid and mitochondrial functional reserve is maintained. In contrast, reductions in SCFA availability, loss of protective indoles, or accumulation of hydrophobic bile acids bias mitochondria toward prolonged states of incomplete recovery, increasing the likelihood that stress is carried forward across successive cycles and triggering quality control–mediated turnover.^103-105^

This comparative view also improves the interpretation of microbiome–mitochondria interactions. A reduction in SCFA signaling would be expected to impair recovery mainly by limiting substrate flux, oxidative metabolism, respiratory reserve, and metabolic flexibility.^26^ ^,^ ^29^ ^,^ ^35^ ^,^ ^36^ ^,^ ^38-45^ A shift in the indole pool would be expected to impair recovery mainly through redox imbalance, membrane instability, altered AhR/NRF2-associated signaling, or accumulation of toxic host-modified indoles.^27^ ^,^ ^56-65^ A dysregulated bile acid pool would be expected to affect recovery through altered TGR5/FXR signaling or, at excessive hydrophobic bile acid levels, through direct mitochondrial membrane damage, electron transport chain impairment, and ROS generation.^16^ ^,^ ^28^ ^,^ ^75^ ^,^ ^81-86^ ^,^ ^90^ ^,^ ^92^ ^,^ ^93^ Therefore, the same mitochondrial phenotype may reflect different microbiome-derived mechanisms depending on which metabolite class is altered.

This convergence reframes microbiome–mitochondria interactions as a process governed not by steady-state activity but by the probability of recovery failure over time. Chemically diverse microbial metabolites can therefore produce similar physiological outcomes across immune, metabolic, and aging-related contexts because they collectively regulate the same underlying recovery processes. At the same time, their mechanistic divergence means that these metabolites are not interchangeable. Rather than acting as isolated signals, microbial metabolites establish the bioenergetic and redox landscape that shapes the probability that repeated challenges are resolved or progressively erode mitochondrial functional reserve.

In this way, direct metabolic signaling from the gut microbiome defines the baseline conditions upon which immune-mediated and stress-related pressures act, linking microbial composition to long-term mitochondrial resilience and cellular vulnerability. This revised interpretation emphasizes both convergence and divergence: SCFAs, indoles, and bile acids converge on mitochondrial recovery capacity, but they do so through distinct and non-redundant mechanisms.

### 2.2 Immune-mediated signaling as sustained mitochondrial pressure

#### 2.2.1. Microbiome-driven immune activation

The gut microbiome continuously shapes baseline immune activity. Importantly, microbiome perturbations rarely induce transient inflammatory responses; instead, they recalibrate immune tone by increasing baseline immune alertness and lowering activation thresholds. This shift produces a chronic low-grade immune state characterized by persistent cytokine production and delayed resolution, even in the absence of overt infection or tissue injury.^106^ ^,^ ^107^

Crucially, this sustained immune tone imposes continuous metabolic and oxidative pressure on mitochondria. Unlike acute inflammatory bursts, which are followed by recovery, microbiome-driven immune activation maintains cells in a prolonged state of low-level stress. Under these conditions, mitochondrial function is not primarily challenged by signal magnitude, but by the persistence of inflammatory demand, which repeatedly engages stress–recovery processes and increases the likelihood of incomplete recovery over time.

One major driver of this state is increased exposure to microbial-associated molecular patterns (MAMPs). Expansion of facultative anaerobes and inflammation-associated taxa including Enterococcus and members of Proteobacteria such as Escherichia—elevates steady-state availability of ligands such as lipopolysaccharide, lipoteichoic acid, and peptidoglycan fragments. Continuous engagement of pattern-recognition receptors on epithelial and myeloid cells sustains low-level production of cytokines including TNF-*α*, IL-1β, and IL-6, maintaining immune cells in a partially activated state.^108^ ^,^ ^109^ This persistent cytokine signaling increases ATP demand, enhances mitochondrial reactive oxygen species production, and constrains the ability of mitochondria to restore redox balance following stress.

At the same time, loss of obligate anaerobic commensals reduces mechanisms that normally restrain immune persistence. Depletion of Clostridiales-associated taxa linked to regulatory immune tone including Faecalibacterium, Roseburia, Eubacterium, and Anaerostipes diminishes microbial signals that support regulatory macrophage and T cell programs.^42^ ^,^ ^49^ Rather than initiating overt inflammation, this shift weakens buffering pathways that typically promote immune resolution once activation has occurred. As a result, mitochondria are exposed to prolonged inflammatory signaling without sufficient recovery intervals, increasing the probability that stress responses are carried forward across successive cycles.

Microbial tryptophan metabolism provides an additional layer of immune modulation. Indole derivatives produced by taxa such as Lactobacillus and Bifidobacterium contribute to immune homeostasis through pathways including aryl hydrocarbon receptor (AhR) signaling. Reduction of these signals does not necessarily initiate inflammation but can prolong cytokine responses and delay immune resolution. In parallel, alterations in mucosal-associated microbial communities increase immune exposure to microbial products. Reduced abundance of mucus-associated commensals, including *Akkermansia muciniphila*, brings microbial signals into closer proximity with the epithelium, increasing immune surveillance pressure even in the absence of overt barrier disruption.^27^ ^,^ ^110^ These changes further reinforce sustained mitochondrial stress and reduce recovery efficiency.

Over time, these pressures increase the likelihood that mitochondrial recovery remains incomplete across repeated stress events, promoting cumulative functional decline and reduced cellular resilience. The aging-specific implications of microbiome-driven immune persistence are integrated with inflammation and immunosenescence in the dedicated aging section below.

Collectively, microbiome imbalance establishes a stable immune environment defined by continuous low-level cytokine output, heightened responsiveness to secondary stimuli, and delayed inflammatory resolution. Although this persistent immune tone rarely produces acute tissue injury, it imposes sustained metabolic and oxidative demands on host cells. Over time, these pressures increase the likelihood that mitochondrial recovery remains incomplete across repeated stress events, promoting cumulative functional decline and reduced cellular resilience during aging.

#### 2.2.2. Immune signaling that reshapes mitochondrial conditions

Chronic immune activation alters the baseline conditions under which mitochondria operate, not by inducing immediate damage, but by persistently constraining the processes required for recovery following stress. Rather than causing acute mitochondrial failure, immune-derived signals impose sustained redox and metabolic pressure that slows recovery dynamics and progressively reduces functional reserve across repeated challenges.^108^ ^,^ ^111^

A central feature of this environment is sustained oxidative and nitrosative stress. Continuous cytokine signaling including TNF-*α*, IL-1β, and IFN-*γ* elevates reactive oxygen and nitrogen species production in both immune and parenchymal cells, increasing basal mitochondrial ROS load and narrowing the redox window within which oxidative phosphorylation can proceed efficiently.^111^ ^,^ ^112^ Under these conditions, restoration of redox balance following stress is delayed, increasing the likelihood that mitochondria remain in partially oxidized states that impair subsequent recovery cycles.

Loss of microbiome-derived immune restraint further amplifies these effects. Reduced availability of short-chain fatty acids and tryptophan-derived indoles is associated with prolonged cytokine signaling and diminished redox buffering capacity.^113^ As a result, mitochondria operate within a chronically oxidizing environment in which recovery of membrane potential and redox equilibrium is less efficient, increasing susceptibility to cumulative oxidative damage across repeated perturbations.

Immune activation also reshapes cellular energy balance in ways that directly constrain recovery. Cytokine exposure elevates ATP demand to support biosynthesis, membrane remodeling, and antioxidant defense, while microbiome alterations that impair fiber fermentation or polysaccharide utilization reduce the stability of metabolic substrate supply.^114^ ^,^ ^115^ This mismatch between energetic demand and substrate availability limits the ability of mitochondria to restore bioenergetic homeostasis following stress, promoting recurrent episodes of incomplete recovery.

In parallel, inflammatory signaling directly impairs mitochondrial respiratory efficiency. Prolonged cytokine exposure is associated with reduced oxygen consumption and decreased electron transport chain efficiency, often accompanied by partial shifts toward glycolytic ATP production. Dysbiosis-associated metabolites can further exacerbate these effects; for example, sulfide-producing taxa generate compounds that interfere with mitochondrial respiration and increase oxidative stress.^116^ ^,^ ^117^ These disruptions reduce the efficiency with which mitochondria recover functional capacity after perturbation.

Sustained immune tone also alters mitochondrial network organization. Increased mitochondrial fragmentation and disruption of fusion–fission balance reduce network connectivity and impair the ability of mitochondria to redistribute metabolic load during stress.^118^ This structural constraint further limits recovery efficiency and increases vulnerability to repeated perturbations.

Collectively, these immune-mediated processes establish a mitochondrial environment characterized by elevated oxidative load, unstable substrate availability, reduced respiratory efficiency, and altered network dynamics. While these conditions rarely eliminate mitochondria directly, they increase the probability that recovery remains incomplete following each stress event. Over time, this leads to the accumulation of partially resolved mitochondrial states, progressive reduction in functional reserve, and increased reliance on quality control pathways, ultimately driving long-term cellular vulnerability during aging.

#### 2.2.3. Persistence of immune pressure under microbiome imbalance

A defining feature of dysbiosis-associated immune activation is its persistence. Unlike acute infection, where pathogen clearance provides a clear termination point, microbiome imbalance sustains immune stimulation under steady-state conditions. This persistence arises from continuous exposure to microbial ligands combined with weakened regulatory and resolution mechanisms.^106^ ^,^ ^119^ Importantly, this sustained immune activity does not simply prolong inflammation, but maintains a chronic upstream pressure that repeatedly challenges mitochondrial recovery processes over time.

First, ongoing innate immune stimulation is maintained by dysbiotic communities enriched in facultative anaerobes such as Proteobacteria and Enterococcus. These taxa increase the baseline availability of microbial-associated molecular patterns (MAMPs), including lipopolysaccharide and peptidoglycan fragments, which engage pattern-recognition receptors on epithelial and myeloid cells. Even in the absence of overt tissue damage, this persistent ligand exposure is sufficient to maintain low-level cytokine production over extended periods. In parallel, depletion of butyrate-producing commensals reduces support for regulatory T cell programs and anti-inflammatory macrophage states, delaying restoration of immune quiescence.^120^ ^,^ ^121^ As a result, mitochondria are exposed to sustained inflammatory signaling without sufficient intervals for recovery, increasing the likelihood that stress responses remain partially unresolved across successive cycles.

Second, adaptive immune set points can become durably altered under specific microbial configurations. Experimental models demonstrate that individual taxa can drive long-lasting shifts in helper T cell polarization for example, segmented filamentous bacteria–induced enrichment of Th17 cells while defined microbial molecules can promote regulatory dominance in other contexts. Dysbiosis that favors pro-inflammatory polarization while diminishing regulatory inputs stabilizes an immune baseline in which inflammatory mediators remain modestly elevated and readily reactivated.^119^ ^,^ ^122^ This persistent immune activation increases the frequency with which mitochondria must respond to inflammatory stress, thereby elevating the probability of incomplete recovery.

Third, alterations in the mucosal interface increase the frequency of immune surveillance. Changes in mucus-associated microbial communities reduce spatial separation between microbes and the epithelium, sustaining epithelial immune sensing even without overt barrier disruption. Loss of mucus-associated commensals such as *Akkermansia muciniphila* has repeatedly been associated with heightened epithelial immune signaling, effectively converting intermittent microbial sensing into continuous immune engagement.^121^ ^,^ ^123^ This increased frequency of immune activation events shortens recovery intervals and promotes accumulation of partially recovered mitochondrial states.

Finally, reduced production of metabolites that promote immune resolution further reinforces immune persistence. Decreased availability of short-chain fatty acids and tryptophan-derived indole ligands weakens resolution pathways mediated by receptors such as AhR and PXR, prolonging cytokine signaling once immune activation has begun.^27^ ^,^ ^42^ In this context, mitochondrial recovery is not only delayed but repeatedly interrupted, further reducing recovery efficiency over time.

Together, continuous innate ligand exposure, durable adaptive immune polarization, altered mucosal spatial organization, and impaired metabolite-driven resolution create a self-reinforcing immune environment. This sustained immune pressure acts as a chronic constraint on mitochondrial recovery, increasing the likelihood that recovery remains incomplete following each stress event. Over time, repeated interruption of recovery processes leads to accumulation of functional deficits and progressive narrowing of mitochondrial reserve, linking microbiome imbalance to long-term cellular vulnerability and aging-associated decline [Table 4].

**Table 4.** Microbiome-driven immune conditions that constrain mitochondrial recovery.

Process/event	Primary driver or condition	Main biological feature	Mitochondrial process affected	Effect on recovery dynamics	Representative references
Continuous innate ligand exposure	Expansion of facultative anaerobes, including Proteobacteria, Enterococcus, and Escherichia	Increased MAMP exposure and persistent PRR signaling	Mitochondrial ROS/RNS burden, redox recovery	Sustains oxidative pressure, delays redox restoration, and increases likelihood of incomplete recovery	Rakoff-Nahoum et al. [124]; Vijay-Kumar et al. [125]
Loss of regulatory commensals	Reduced Faecalibacterium, Roseburia, Eubacterium, and Anaerostipes	Reduced SCFA-mediated immune restraint and weakened regulatory buffering	Redox buffering, inflammatory resolution, oxidative stress tolerance	Prolongs inflammatory stress and reduces mitochondrial recovery efficiency	Furusawa et al. [42]; Arpaia et al. [126]
Reduced tryptophan–indole signaling	Reduced Lactobacillus and Bifidobacterium or altered indole pool	Decreased AhR-associated immune regulation and antioxidant signaling	Redox balance, membrane potential recovery, inflammatory amplification	Delays resolution of inflammatory stress and increases recovery burden	Zelante et al. [27]; Lamas et al. [127]
Stable adaptive immune skewing	Specific taxa such as SFB or loss of regulatory microbial signals	Altered Th17/Treg balance and fixed immune set point	Frequency of immune-mediated mitochondrial stress	Repeatedly interrupts recovery and increases cumulative recovery failure	Ivanov et al. [122]; Honda and Littman [107]
Altered mucosal spatial organization	Loss or reduction of mucus-associated commensals such as *Akkermansia muciniphila*	Increased epithelial immune sensing and immune surveillance	Subthreshold stress frequency, epithelial mitochondrial load	Shortens recovery intervals and promotes accumulation of partially recovered mitochondrial states	Johansson et al. [128]; Everard et al. [51]
Persistent cytokine signaling	Dysbiosis-associated low-grade immune activation	Sustained TNF-*α*, IL-1β, IL-6, and IFN-*γ* signaling	ROS/RNS burden, ETC efficiency, ATP demand	Increases energetic strain and delays restoration of redox and bioenergetic balance	West et al. [111]; Brown and Borutaite [113]
Elevated biosynthetic and antioxidant demand	Chronic immune activation with unstable substrate supply	Increased ATP demand for immune activation and stress responses	ATP demand–supply balance, TCA input stability	Promotes recovery failure through persistent demand–output mismatch	Pearce and Pearce [115]; Den Besten et al. [35]
Dysbiosis-associated respiratory inhibition	Expansion of sulfide-producing or inflammation-associated taxa	Production of metabolites that interfere with mitochondrial respiration	Respiratory chain integrity, oxygen consumption efficiency	Impairs recovery capacity by reducing respiratory efficiency and increasing oxidative stress	Furne et al. [116]; Wallace and Wang [117]
Altered mitochondrial network dynamics	Sustained inflammatory tone	Increased mitochondrial fragmentation and reduced network connectivity	Fusion–fission balance, mitochondrial network dynamics	Reduces recovery efficiency by limiting redistribution of metabolic load during stress	Youle and van der Bliek [129]
Integrated chronic immune pressure	Combined dysbiosis, delayed immune resolution, and chronic low-grade inflammation	Persistent immune activation without acute injury	Functional reserve, mitochondrial recovery capacity	May contribute to cumulative recovery impairment and progressive reduction in mitochondrial reserve	Belkaid and Hand [106]; Picard et al. [130]

### 2.3. Cellular stress pathways linking microbiome-driven signals to mitochondrial quality control

#### 2.3.1. Persistent functional stress as a signal of mitochondrial inadequacy

Mitochondria are not eliminated in response to isolated or transient stress. Instead, mitochondrial turnover is preferentially engaged when functional disturbances recur or persist beyond the cell’s ability to restore stable activity. Under conditions of microbiome imbalance, the metabolic and immune pressures described in Sections 2.1 and 2.2 increase the likelihood that mitochondrial stress becomes sustained rather than episodic. In this context, mitochondrial dysfunction is not defined by acute damage, but by repeated failure to fully recover following perturbation.

One sensitive indicator of this process is prolonged elevation of mitochondrial reactive oxygen species (ROS). Under physiological conditions, ROS generated during oxidative phosphorylation are efficiently neutralized, allowing redox balance to be restored after transient fluctuations. However, reduced availability of short-chain fatty acids and tryptophan-derived indoles weakens redox buffering capacity, while persistent immune signaling elevates basal oxidative and nitrosative pressure. Together, these factors promote sustained ROS accumulation that delays restoration of redox equilibrium following stress.^118^ ^,^ ^131^ As a result, mitochondria are more likely to remain in partially oxidized states, increasing the probability that recovery remains incomplete across successive cycles.

Stability of the mitochondrial membrane potential (ΔΨm) provides a second critical signal of mitochondrial adequacy. Maintenance of ΔΨm is essential for ATP synthesis, protein import, and ionic homeostasis. In environments shaped by chronic cytokine exposure, recovery of membrane potential following depolarization becomes slower and less complete. Repeated episodes of incomplete ΔΨm restoration reflect declining efficiency of electron transport and reduced tolerance to metabolic fluctuations.^103^ ^,^ ^104^ These partially repolarized states represent a key intermediate in which mitochondria remain functional but fail to fully recover, predisposing them to subsequent stress.

A third signal arises from recurrent imbalance between ATP supply and cellular energy demand. Sustained immune activation increases energetic requirements for biosynthesis, membrane remodeling, and stress responses, while microbiome-associated alterations reduce substrate availability and metabolic flexibility. Under these conditions, mitochondria may repeatedly fail to meet peak energetic demands. Importantly, this mismatch is often intermittent rather than continuous, generating cycles of energetic strain that do not fully resolve.^18^ ^,^ ^132^ Each cycle of unmet demand further constrains recovery capacity and increases susceptibility to subsequent perturbations.

Taken together, persistent ROS elevation, delayed recovery of membrane potential, and recurrent energetic shortfall do not simply indicate mitochondrial stress; they reflect a progressive increase in the probability of recovery failure. These features signal that mitochondrial recovery capacity is becoming constrained, even in the absence of overt structural damage. Over time, accumulation of incompletely resolved stress events promotes activation of mitochondrial quality control pathways, linking microbiome-driven metabolic and immune pressures to selective mitochondrial turnover [Table 5].

**Table 5.** Mitochondrial recovery failure, quality-control engagement, and loss of functional reserve.

Process/event	Primary driver or condition	Main biological feature	Mitochondrial process affected	Effect on recovery dynamics	Representative references
Recurrent stress exposure	Chronic immune activation and repeated metabolic or oxidative perturbation	Repeated mitochondrial functional challenge	Initiation of incomplete recovery across stress–recovery cycles	Increases the probability that stress is carried forward into subsequent cycles	Picard et al. [100]; West et al. [133]
Sustained mitochondrial ROS	Redox buffering exceeded under inflammatory or oxidative pressure	Redox balance, ETC stability	Permissive mitophagy signaling and oxidative damage accumulation	Delays redox recovery and increases likelihood of recovery failure	West et al. [111]; Murphy et al. [131]
Chronic cytokine/NO exposure	Persistent TNF-*α*, IL-1β, IFN-*γ*, or nitric oxide signaling	ETC activity, mitochondrial ROS/RNS burden	Redox margin narrowing and reduced respiratory efficiency	Sustains oxidative constraints that slow mitochondrial recovery	Brown and Borutaite [113]; Victor et al. [134]
Delayed ΔΨm restoration	Repeated or prolonged mitochondrial depolarization	Mitochondrial membrane potential	PINK1 stabilization and Parkin recruitment	Reflects failure to fully restore membrane potential after stress	Narendra et al. [12]; Matsuda et al. [135]
Prolonged depolarized state	Sustained ΔΨm loss or incomplete repolarization	Outer mitochondrial membrane quality-control signaling	Ubiquitin signaling and mitophagy initiation	Signals unresolved recovery failure leading to selective mitochondrial removal	Lazarou et al. [136]
Loss of stabilizing metabolites	Reduced butyrate and protective indole availability	Redox buffering, ΔΨm repolarization, respiratory efficiency	Prolonged recovery phase and increased mitochondrial vulnerability	Increases incomplete recovery by weakening metabolic and redox support	Donohoe et al. [26]; Zelante et al. [27]
Recurrent ATP shortfall	Energetic demand exceeds mitochondrial output	ATP-linked respiration, ATP demand–supply balance	AMPK activation, ULK1 phosphorylation, mitophagy priming	Reinforces recovery failure through repeated energetic mismatch	Egan et al. [137]; Toyama et al. [138]
Bioenergetic heterogeneity	Mixed mitochondrial population under chronic stress	Mitochondrial population quality and fission–fusion balance	Fission of low-function mitochondria and selective removal	Segregates mitochondria unable to recover efficiently	Twig et al. [139]; Youle and van der Bliek [129]
Basal preserved/reserve reduced state	Preserved basal output with reduced maximal capacity	Basal ATP production, maximal respiration, SRC	Functional reserve narrowing	Maintains resting energy output but reduces recovery capacity and functional reserve	Brand and Nicholls [18]; Larsen et al. [140]; Desler et al. [141]; Short et al. [142]
Delayed recovery after depolarization	Stress condition following reversible mitochondrial depolarization	ΔΨm recovery kinetics	Prolonged recovery time and increased vulnerability to repeated stress	Indicates reduced recovery capacity despite possible preservation of basal function	Narendra et al. [12]; Scaduto and Grotyohann [104]
Increased oxidative sensitivity	Elevated oxidative stress under repeated challenge	Redox buffering, mitochondrial ROS resolution	Lower threshold for recovery failure	Increases probability of incomplete recovery under oxidative conditions	Murphy et al. [131]
Impaired response to increased workload	Additional metabolic or inflammatory demand	Maximal respiration, SRC, metabolic flexibility	Reduced recovery capacity	Limits the ability to recover from increased energetic demand	Sohal et al. [143]
Incomplete maturation of new mitochondria	Biogenesis under persistent metabolic or inflammatory stress	Mitochondrial maturation and functional optimization	Newly generated mitochondria show reduced recovery capacity	Reinforces long-term decline in mitochondrial flexibility	Ploumi et al. [144]; Picard et al. [130]
Chronic recovery failure	Repeated incomplete stress resolution	Mitochondrial population profile, functional reserve	Increased quality-control engagement and progressive reserve loss	Drives cumulative functional decline and long-term vulnerability	Cordeiro et al. [145]; Picard et al. [130]

#### 2.3.2. From impaired recovery to mitochondrial turnover

When functional stress signals persist, mitochondrial fate is determined not by the severity of individual insults, but by the ability to restore function between successive challenges. Under conditions of adequate metabolic support and redox control, transient disturbances are resolved through recovery of electron transport efficiency, membrane potential, and ATP production. In contrast, microbiome-driven immune activation and metabolite imbalance weaken these recovery processes, increasing the probability that recovery remains incomplete across repeated stress–recovery cycles.^104^ ^,^ ^111^ ^,^ ^131^

Sustained oxidative and nitrosative pressure interferes with electron transport chain activity and delays restoration of redox balance. At the same time, reduced availability of stabilizing microbial metabolites including butyrate and protective indoles slows repolarization of the mitochondrial membrane and limits recovery of respiratory efficiency.^113^ ^,^ ^131^ As a result, mitochondria remain in partially recovered states following each perturbation, creating a persistent functional deficit that carries forward into subsequent cycles.

As these conditions persist, mitochondrial quality-control pathways are more frequently engaged to remove mitochondria that fail to restore membrane potential, redox balance, or respiratory efficiency. Whether this response preserves mitochondrial quality or contributes to functional decline depends on the completeness of mitophagy flux and the capacity for mitochondrial replacement. Prolonged membrane depolarization and unresolved oxidative stress promote accumulation of quality control signals on the mitochondrial surface, including stabilization of PINK1 and recruitment of Parkin, thereby increasing engagement of mitophagy-related mechanisms. Importantly, this transition does not require catastrophic mitochondrial injury; rather, it reflects repeated failure to re-establish stable mitochondrial function.^26^ ^,^ ^57^ Importantly, mitophagy engagement should not be interpreted as intrinsically pathological. PINK1/Parkin-mediated mitophagy is an adaptive quality-control pathway that preserves mitochondrial population quality when damaged or poorly polarized mitochondria are selectively removed and replacement through mitochondrial biogenesis and functional maturation is sufficient. Pathological consequences emerge only when quality-control balance is disrupted. Three scenarios should therefore be distinguished. First, adaptive mitophagy can support recovery by eliminating damaged organelles while maintaining mitochondrial mass and functional reserve through adequate replacement. Second, excessive mitophagy can reduce mitochondrial content and narrow functional reserve when mitochondrial removal exceeds biogenesis or when newly generated mitochondria fail to mature fully. Third, impaired mitophagy flux can allow damaged mitochondria to accumulate when initiation signals such as PINK1 stabilization or Parkin recruitment are not followed by efficient lysosomal clearance. Thus, mitochondrial decline is not caused by mitophagy activation per se, but by imbalance among recovery efficiency, mitophagy flux, mitochondrial biogenesis, and functional replacement.

In parallel, immune-driven increases in energetic demand can exceed mitochondrial recovery capacity. Recurrent ATP insufficiency during periods of immune or environmental stress biases cells toward preferential elimination of less efficient mitochondria. Across repeated stress–recovery cycles, this selective pressure gradually reshapes the mitochondrial population, enriching for mitochondria capable of maintaining function under constrained conditions while eliminating those that repeatedly fail to recover.

Over time, this process may be associated with a progressive reduction in mitochondrial functional reserve without abrupt loss of mitochondrial mass. The cumulative effect of repeated incomplete recovery and selective turnover is a mitochondrial population that is increasingly constrained in its recovery capacity and functional reserve. This framework proposes a testable link between microbiome-associated metabolic and immune pressures and long-term changes in mitochondrial composition, providing a mechanistic basis for gradual functional decline and increased vulnerability to stress during aging.

#### 2.3.3. Reduced mitochondrial recovery capacity across repeated stress–recovery cycles

Repeated exposure to microbiome-driven immune and metabolic stress does not typically result in abrupt mitochondrial failure. Instead, mitochondrial function may become progressively constrained through a gradual reduction in recovery efficiency across repeated challenges. Under these conditions, mitochondria retain basal energetic output but lose the capacity to respond flexibly to increased metabolic or inflammatory demand.^141^

This transition is most clearly reflected in the dissociation between basal respiration and maximal respiratory capacity. Basal ATP production and ATP-linked oxygen consumption can remain largely preserved, even as maximal respiratory capacity and spare respiratory capacity (SRC) decline. This pattern represents a state in which cells operate closer to their bioenergetic ceiling, with limited capacity to accommodate additional stress.^146^ From a recovery-centered perspective, reduced SRC reflects diminished recovery bandwidth, defined as the ability of mitochondria to restore function following perturbation, rather than simple impairment of steady-state energy production.

To measure this process more directly, mitochondrial recovery should be evaluated using kinetic and repeated-measurement assays rather than endpoint measurements alone.^18-20^ Useful parameters include ΔΨm repolarization kinetics after reversible depolarization,^12^ ^,^ ^104^ NAD⁺/NADH restoration after metabolic or oxidative challenge, recovery of ATP-linked respiration, SRC rebound after a recovery period, and mitophagy flux based on PINK1/Parkin recruitment, LC3 colocalization with mitochondria, or lysosomal turnover of mitochondrial proteins.^12^ ^,^ ^13^ ^,^ ^135^ ^,^ ^136^ These readouts would help distinguish impaired recovery capacity from reduced basal mitochondrial activity.

Primary studies in systemic inflammatory settings support this interpretation. In pediatric sepsis, peripheral blood mononuclear cells exhibit early loss of bioenergetic reserve, including reduced maximal electron transfer system capacity (ETS_max) and SRC, while maintaining near-normal ATP-linked respiration at presentation. Similarly, during aging-associated immune remodeling, classical monocytes from older adults display reduced total respiratory capacity and diminished SRC despite preserved baseline energetic output.^146^ These observations indicate that mitochondrial dysfunction in these contexts is more consistently expressed as reduced recovery capacity and impaired stress recovery than as immediate energetic failure.

Importantly, this “basal preserved/reserve reduced” pattern is context-dependent and varies with cell type, normalization strategy, and temporal phase of the response. In some settings, including early recovery phases of sepsis, mitochondrial respiratory capacity may transiently increase, reflecting compensatory biogenesis and increased mitochondrial content. However, even in these cases, mitochondrial adaptation occurs within a constrained environment shaped by persistent immune and metabolic pressure.^18^ ^,^ ^130^ This reinforces a recovery-centered framework in which the dominant effect of microbiome-related perturbation is not the direction of change in mitochondrial activity, but the narrowing of recovery capacity and functional reserve over time.

Microbiome imbalance sustains this trajectory by continuously biasing the cellular environment against efficient mitochondrial recovery. Persistent immune signaling elevates oxidative and nitrosative pressure, while reduced availability of short-chain fatty acids and tryptophan-derived indoles limits metabolic flexibility and redox stabilization. Consequently, newly generated mitochondria mature within an environment that restricts full functional optimization, predisposing them to incomplete recovery following subsequent stress.^14^ ^,^ ^147^

Over time, this selective pressure reshapes the mitochondrial population. Mitochondria capable of maintaining basal function are retained, whereas those unable to recover efficiently from repeated perturbations are preferentially removed. The resulting population is optimized for steady-state maintenance but shows reduced recovery flexibility. This shift reduces mitochondrial recovery capacity at the population level and increases vulnerability to secondary stressors, including immune activation, metabolic strain, and age-associated physiological decline.^129^ ^,^ ^148^

**Figure 1.**
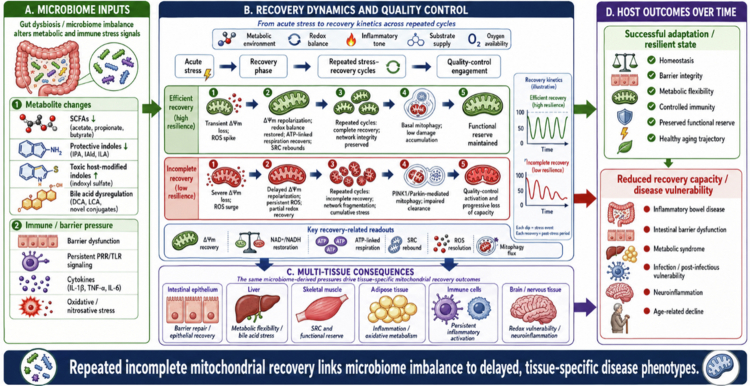
Microbiome-driven pressures bias mitochondria toward repeated recovery failure and quality control engagement. (A) Gut dysbiosis alters microbial metabolites and immune/barrier signals, including reduced SCFAs and protective indoles, increased toxic host-modified indoles, bile acid dysregulation, cytokine signaling, and oxidative or nitrosative stress. (B) These metabolic, redox, inflammatory, substrate, and oxygen-related pressures shape mitochondrial recovery after stress. Efficient recovery involves ΔΨm repolarization, ROS resolution, redox restoration, ATP-linked respiration recovery, SRC rebound, basal mitophagy, and maintained functional reserve. Incomplete recovery involves delayed ΔΨm restoration, persistent ROS, partial redox recovery, mitochondrial network fragmentation, increased quality-control engagement, and reduced recovery capacity. (C) The same microbiome-derived pressures may produce tissue-specific outcomes in the intestinal epithelium, liver, skeletal muscle, adipose tissue, immune cells, and brain/nervous tissue. (D) Efficient recovery may support resilient host states, whereas repeated incomplete recovery may increase vulnerability to inflammatory, metabolic, infectious, neuroinflammatory, and age-related disease phenotypes.

Because immune and metabolic signals propagate systemically, similar constraints on mitochondrial recovery capacity can emerge across multiple tissues, even when the initiating perturbation originates in the gut. In this way, microbiome imbalance contributes to a gradual, organism-wide reduction in mitochondrial resilience and tissue-level vulnerability to repeated stress, linking local microbial dysregulation to systemic vulnerability without requiring direct infection or acute tissue damage [Figure 1].

### 2.4. Aging, inflammaging, and microbiome-dependent mitochondrial recovery

Taken together, the mechanisms described in Sections 2.1–2.3 support a unified framework in which the gut microbiome regulates mitochondrial function primarily by shaping the conditions that determine recovery following stress, rather than by directly imposing static changes in mitochondrial activity. Microbial metabolites influence substrate availability, redox buffering, membrane stability, and metabolic signaling, while microbiome-driven immune activation imposes sustained oxidative and energetic pressure.^26-28^ ^,^ ^56-67^ ^,^ ^75^ ^,^ ^81-86^ ^,^ ^92^ ^,^ ^93^ ^,^ ^111-113^ These factors converge to define the environment within which mitochondrial stress responses are initiated, resolved, or persist.

Within this framework, mitochondrial quality control is engaged not by isolated perturbations, but by the persistence and recurrence of functional stress that fails to fully resolve. Repeated disturbances that do not return to baseline increase the burden on mitochondrial maintenance systems and shift the balance among functional restoration, selective turnover, and mitochondrial replacement.^12^ ^,^ ^13^ ^,^ ^135^ ^,^ ^136^ ^,^ ^144^ As a result, mitochondrial dynamics are governed by the probability of recovery failure over time, leading to gradual remodeling of mitochondrial populations rather than abrupt mitochondrial loss.^100^ ^,^ ^130^

This model does not imply that mitophagy is uniformly detrimental. Under physiological conditions, mitophagy is essential for mitochondrial quality maintenance and can support recovery by removing damaged mitochondria before they propagate oxidative or energetic stress.^12^ ^,^ ^13^ ^,^ ^135^ ^,^ ^136^ However, microbiome-driven metabolic and immune pressure may shift this balance in two maladaptive directions. If mitophagy initiation is excessive and is not matched by mitochondrial biogenesis and functional maturation, mitochondrial mass and functional reserve may decline. Conversely, if mitophagy flux is impaired, damaged mitochondria may persist, increasing ROS production and lowering recovery efficiency.^131^ ^,^ ^135^ ^,^ ^136^ ^,^ ^144^ Therefore, mitochondrial resilience depends not only on whether mitophagy is activated, but on whether mitophagy flux, mitochondrial biogenesis, and functional maturation remain coordinated.^130^ ^,^ ^144^

Aging provides a disease-relevant context in which this recovery-centered process may become amplified. Age-related microbiome shifts, inflammation, and immunosenescence are related but distinct processes.^21-25^ ^,^ ^149^ ^,^ ^150^ Age-related microbiome shifts include reduced abundance of butyrate-producing taxa, altered tryptophan-derived indole metabolism, and changes in bile acid transformation, which together may weaken substrate support, redox buffering, and receptor-mediated metabolic signaling.^23-28^ ^,^ ^56-67^ ^,^ ^75^ ^,^ ^81-86^ ^,^ ^92^ ^,^ ^93^ Inflammation refers to chronic low-grade inflammatory tone that increases oxidative, nitrosative, and energetic pressure on mitochondria.^111-113^ ^,^ ^149^ ^,^ ^150^ Immunosenescence reflects reduced immune response flexibility and delayed resolution after stimulation, which can prolong cytokine exposure and shorten recovery intervals.^149^ ^,^ ^150^ Together, these processes may lower mitochondrial recovery capacity by delaying restoration of ΔΨm, redox balance, ATP-linked respiration, and SRC after stress.^18-20^ ^,^ ^142^ ^,^ ^151^ In this view, aging acts not as a single mechanism but as a modifier of recovery thresholds, explaining why similar microbiome-derived perturbations may produce stronger or more persistent phenotypes in older hosts.^21-25^ ^,^ ^152^ ^,^ ^153^

This perspective provides a mechanistic explanation for several defining features of microbiome-associated phenotypes. First, mitochondrial consequences typically emerge gradually, reflecting the cumulative effects of repeated incomplete recovery rather than acute damage.^100^ ^,^ ^130^ Second, because immune and metabolic signals propagate systemically, constraints on mitochondrial recovery can arise across multiple tissues even when the initiating perturbation originates in the gut.^106^ ^,^ ^111^ ^,^ ^113^ ^,^ ^134^ Third, variability in host metabolic buffering capacity and immune responsiveness determines how effectively recovery processes are maintained, allowing similar microbial configurations to produce divergent physiological outcomes across individuals.^4^ ^,^ ^5^ ^,^ ^152^ ^,^ ^153^

Viewed through this lens, the gut microbiome does not dictate fixed mitochondrial states, but instead modulates the likelihood that mitochondrial recovery succeeds or fails under repeated challenge. Over time, this bias toward incomplete recovery leads to progressive reduction in functional reserve and increased burden on mitochondrial quality-control pathways.^12^ ^,^ ^13^ ^,^ ^130^ ^,^ ^135^ ^,^ ^136^ ^,^ ^144^ This recovery-centered framework links microbiome imbalance to long-term shifts in mitochondrial function, providing a unifying explanation for the delayed, systemic, and context-dependent nature of microbiome-associated disease vulnerability.^4^ ^,^ ^5^ ^,^ ^152^ ^,^ ^154^

## 3. Discussion

Despite extensive characterization of gut microbial composition and metabolite profiles, a central challenge remains unresolved: how microbiome-derived signals are converted into stable, long-term host phenotypes. Associations between specific taxa or metabolites and immune, metabolic, or neurological outcomes are well established, yet these relationships frequently lack temporal precision and predictive consistency.^152^ ^,^ ^154^ Similar microbial perturbations can produce divergent outcomes across individuals and tissues, and disease-associated phenotypes often emerge gradually rather than following discrete microbial shifts. These observations suggest that microbiome-derived signals are not interpreted as isolated events, but as time-integrated pressures that accumulate within host systems.^155^

In this review, we propose that the missing integrative layer linking microbiome activity to host physiology lies in mitochondrial recovery dynamics and the threshold-dependent engagement of mitochondrial quality control. Rather than being governed only by the magnitude of individual signals, such as peak cytokine levels or metabolite concentrations, mitochondrial responses are strongly influenced by the persistence and recurrence of functional stress. In this framework, cellular outcomes are shaped by whether mitochondria can repeatedly restore functional stability following perturbation, rather than by the intensity of any single insult.

This recovery-centered framework differs from conventional signal-centric models in both explanatory focus and experimental prediction. Signal-centric models are valuable for identifying microbial metabolites, cytokines, receptors, and downstream pathways that acutely modify host physiology. However, they generally assume that biological outcomes scale with signal identity or magnitude at a given time point. This logic is less able to explain why similar microbiome configurations can produce delayed, heterogeneous, or tissue-specific phenotypes. In contrast, the recovery-centered model emphasizes signal persistence, recurrence, and the ability of mitochondria to restore function after repeated perturbation. It therefore predicts that disease vulnerability will be better explained by recovery kinetics, including ΔΨm restoration, NAD⁺/NADH rebalance, ATP-linked respiration recovery, SRC rebound, ROS resolution, and mitophagy flux, than by basal mitochondrial activity or metabolite abundance alone.

At the mechanistic level, mitochondrial quality control behaves as a history-dependent process that preferentially responds to unresolved recovery. Stabilization of PINK1 on the outer mitochondrial membrane requires sustained or recurrent loss of membrane potential, and subsequent Parkin recruitment and mitophagy are favored under conditions of persistent oxidative stress and repeated energetic insufficiency.^103^ ^,^ ^156^ In contrast, transient increases in reactive oxygen species or short-lived ATP imbalance are often resolved through redox buffering and metabolic compensation without commitment to turnover. This distinction establishes recovery failure, rather than damage per se, as a critical signal that can drive mitochondrial selection.

The gut microbiome is uniquely positioned to generate this pattern of persistent functional pressure. Microbiome-derived metabolites, including short-chain fatty acids, tryptophan-derived indoles, and microbiota-modified bile acids, define substrate availability, redox buffering capacity, membrane stability, receptor-mediated metabolic signaling, and transcriptional control of mitochondrial metabolism.^34^ ^,^ ^57^ At the same time, microbiome-driven immune activation imposes sustained oxidative and energetic demand.^134^ ^,^ ^157^ Individually, these signals are often modest; collectively, their persistence converts them into continuous constraints on mitochondrial recovery. Under these conditions, mitochondria are repeatedly pushed into states of incomplete recovery, increasing the probability that functional deficits accumulate across successive cycles.

This framework provides a mechanistic explanation for a recurring and otherwise paradoxical observation across microbiome-associated conditions: preservation of basal ATP production alongside reduced maximal respiratory capacity, impaired recovery following stress, and heightened sensitivity to secondary challenges.^100^ ^,^ ^158^ Rather than inducing overt mitochondrial failure, microbiome imbalance narrows the adaptive range within which mitochondria can operate. Cells may remain viable under resting conditions, yet progressively lose the capacity to accommodate additional metabolic or inflammatory demand. This progressive constraint offers a plausible basis for delayed disease onset and the amplification of phenotypes during aging, when mitochondrial reserve and recovery capacity are already diminished.^159^ ^,^ ^160^

Inter-individual variability also emerges naturally from this model. Differences in baseline mitochondrial buffering capacity, efficiency of redox control, immune responsiveness, and quality-control thresholds determine whether persistent microbiome-derived pressure remains compensable or becomes cumulative.^109^ ^,^ ^161^ As a result, similar microbial configurations may produce markedly different physiological outcomes depending on host context, helping to explain the limited predictive power of microbiome-based biomarkers when considered independently of host physiology.

Several host factors may determine these individual differences. Genetic background and baseline host physiology can influence mitochondrial respiration, antioxidant defense, inflammatory responses, and mitophagy activity.^4^ ^,^ ^5^ ^,^ ^161^ Age can reduce mitochondrial reserve and slow recovery after stress, making older hosts more vulnerable to repeated microbiome-derived pressure.^21^ ^,^ ^22^ ^,^ ^142^ ^,^ ^151^ Sex- or gender-associated differences in immune activity, hormone signaling, body composition, and mitochondrial substrate use may also affect recovery capacity.^153^ Diet is another major factor because it shapes microbial metabolite production, nutrient availability, NAD⁺ metabolism, bile acid composition, and systemic inflammation.^4^ ^,^ ^14^ ^,^ ^16^ ^,^ ^28^ ^,^ ^35^ Disease status, including obesity, diabetes, inflammatory disease, infection, and neurodegenerative disease, may further reduce mitochondrial recovery by adding pre-existing oxidative, metabolic, or immune stress.^1^ ^,^ ^2^ ^,^ ^8^ ^,^ ^9^ ^,^ ^21^ ^,^ ^22^ These factors may explain why similar microbiome compositions do not always produce the same host phenotype.^4^ ^,^ ^5^ ^,^ ^152^ ^,^ ^154^

This recovery-centered perspective has important implications for therapeutic strategies. Interventions that focus solely on restoring individual taxa or acutely suppressing inflammatory signaling may fail to restore mitochondrial recovery capacity if the underlying constraints on recovery persist. In contrast, approaches that enhance metabolic support, stabilize redox environments, preserve physiological metabolite signaling, strengthen epithelial barrier function, and promote immune resolution are more likely to restore mitochondrial flexibility over time.^161^ ^,^ ^162^ The requirement for sustained intervention aligns with clinical observations that microbiome-targeted therapies often require prolonged administration to achieve durable benefit.

The translational relevance of this model is most apparent in diseases where microbiome imbalance, immune activation, epithelial dysfunction, and mitochondrial stress intersect. In inflammatory bowel disease, longitudinal microbiome instability, reduced microbial metabolic support, persistent mucosal cytokine signaling, and impaired epithelial energetics may limit mitochondrial recovery in the intestinal epithelium and immune compartment.^8^ ^,^ ^9^ Intestinal barrier dysfunction can be interpreted through a similar mechanism, in which reduced butyrate-dependent epithelial oxidative metabolism, altered mucus-associated microbial communities, and increased immune sensing shorten recovery intervals and weaken barrier restoration.^26^ ^,^ ^32-34^ ^,^ ^50^ ^,^ ^51^ ^,^ ^55^ In metabolic syndrome, altered SCFA production, dysregulated bile acid signaling, chronic low-grade inflammation, and impaired substrate flexibility may reduce mitochondrial functional reserve in liver, adipose tissue, skeletal muscle, and immune cells.^1^ ^,^ ^2^ ^,^ ^14^ ^,^ ^16^ ^,^ ^28^ In infection or post-infectious states, persistent microbial ligand exposure and delayed immune resolution may prolong mitochondrial oxidative stress even after pathogen burden decreases.^10^ ^,^ ^111^ ^,^ ^113^ These disease contexts suggest that mitochondrial recovery capacity may serve as a functional readout linking microbiome disturbance to disease persistence, relapse, or incomplete recovery.

Recent human clinical and multi-omics studies further strengthen the translational relevance of this framework. In inflammatory bowel disease, large-scale metagenomic and metabolomic analyzes have shown that microbiome and metabolite perturbations are consistently associated with disease status, progression, and treatment-relevant host responses, supporting the need to move beyond single-taxon associations toward functional microbiome readouts.^152^ ^,^ ^154^ Recent studies have also demonstrated the potential of microbiome-based diagnostic models in IBD and have shown that microbial metabolism can influence therapeutic response, including the metabolism of 5-aminosalicylic acid.^163^ ^,^ ^164^ In systemic disease contexts, gut microbiome-derived indoxyl sulfate has recently been linked to chronic kidney disease-associated heart failure in humans, with mechanistic evidence connecting indoxyl sulfate to mitochondrial dysfunction through the AHR-CYP1B1 axis.^165^ Although these studies do not yet directly measure mitochondrial recovery kinetics, they support the clinical relevance of microbiome-derived metabolites as modifiers of host metabolic and inflammatory phenotypes.

This framework also suggests actionable intervention strategies. Restoring SCFA-producing microbial networks, enhancing protective microbiota-derived indoles, reducing toxic host-modified indoles, correcting dysregulated bile acid pools, strengthening epithelial barrier function, and promoting immune resolution may improve mitochondrial recovery capacity. Therapeutic efficacy should therefore be evaluated using recovery-oriented endpoints such as SRC rebound, ΔΨm restoration, NAD⁺/NADH recovery, mitochondrial ROS resolution, ATP-linked respiration recovery, and mitophagy flux.^18-20^ ^,^ ^104^ ^,^ ^135^ ^,^ ^136^ Such endpoints could help determine whether microbiome-targeted interventions restore mitochondrial recovery capacity rather than only changing microbial composition or suppressing inflammation.

However, several points require careful interpretation. Much of the current evidence linking microbial metabolites to mitochondrial outcomes comes from endpoint measurements, acute treatment experiments, or disease-associated comparisons. These studies support mechanistic plausibility, but they do not fully establish whether microbiome-derived signals directly cause progressive recovery failure across repeated stress–recovery cycles. Mitochondrial responses to stress are also not uniformly suppressive. In some settings, mitochondrial respiration, mitochondrial biogenesis, or spare respiratory capacity may transiently increase as part of compensatory adaptation. Therefore, reduced mitochondrial activity should not be assumed to be the universal outcome of microbiome imbalance.

The weight of evidence supporting this framework should be interpreted at two levels. The strongest evidence supports individual mechanistic components: microbial metabolites regulate epithelial mitochondrial metabolism and oxygenation,^26^ ^,^ ^32-34^ microbiota-derived indole signals modulate redox, barrier, and immune pathways,^27^ ^,^ ^57-60^ ^,^ ^63-65^ and sustained mitochondrial depolarization can engage PINK1/Parkin-mediated quality control.^12^ ^,^ ^13^ ^,^ ^135^ ^,^ ^136^ A second, more inferential level links these components into a longitudinal recovery model. Direct evidence for microbiome-driven mitochondrial recovery dynamics remains limited because most existing studies were not designed to measure serial stress–recovery cycles. Thus, the recovery-centered framework is best viewed as a mechanistically grounded and experimentally testable model, rather than as a fully established causal pathway.

Potentially conflicting findings should be interpreted according to timing, cell type, normalization strategy, and disease phase. Early compensatory increases in respiratory capacity may reflect mitochondrial biogenesis or increased mitochondrial content, whereas later reductions in spare respiratory capacity may reflect narrowing of functional reserve. Similarly, microbial metabolites should not be interpreted as uniformly beneficial or harmful. SCFAs may support oxidative metabolism and metabolic flexibility, but may not fully restore recovery if inflammatory pressure persists. Microbiota-derived indoles may stabilize redox balance when present as protective microbiota-derived metabolites, whereas host-modified indoles such as indoxyl sulfate may impair mitochondrial recovery. Microbiota-modified bile acids may support metabolic signaling through TGR5 and FXR within physiological ranges, but excessive hydrophobic bile acid accumulation may directly damage mitochondrial membranes and impair respiration. These considerations indicate that microbiome–mitochondria interactions are context-dependent and should be evaluated using time-resolved and tissue-specific approaches.

Several limitations should therefore be acknowledged. Direct longitudinal studies linking defined microbiome perturbations to mitochondrial recovery kinetics and quality-control engagement remain limited, particularly across multiple tissues. The proposed sequence from microbiome-driven metabolic or immune stress to incomplete mitochondrial recovery and mitochondrial quality-control engagement should therefore be interpreted as a testable model rather than a fully established causal pathway. Few studies have simultaneously measured microbiome composition, microbial metabolites, immune activation, ΔΨm recovery kinetics, NAD⁺/NADH restoration, spare respiratory capacity rebound, and mitophagy flux over time. Tissue-specific differences in immune exposure, metabolic demand, mitochondrial turnover, and host detoxification capacity are also likely to influence outcomes. In addition, while this review emphasizes mitochondrial recovery, parallel regulatory systems, including neuroendocrine signaling, epigenetic adaptation, and organ-specific metabolic remodeling, may interact with mitochondrial processes in context-dependent ways.

To directly test the recovery-centered model, future microbiome–mitochondria studies should move beyond single endpoint measurements and adopt standardized longitudinal stress–recovery designs.^8^ ^,^ ^9^ ^,^ ^18-20^ A practical design may include four sequential phases: baseline profiling, defined perturbation, recovery monitoring, and repeated challenge. Such studies could combine defined microbial communities, fecal microbiota transplantation, or individual microbial metabolites with repeated metabolic, inflammatory, or oxidative challenges, followed by synchronized microbiome, metabolite, immune, and mitochondrial measurements across baseline, stress, recovery, and repeated-challenge phases.^8^ ^,^ ^9^ ^,^ ^18-20^ In this design, serial stress–recovery respirometry would quantify basal respiration, ATP-linked respiration recovery, maximal respiration, and SRC rebound after each challenge.^18-20^ Live-cell ΔΨm imaging would measure repolarization kinetics after repeated reversible depolarization.^12^ ^,^ ^104^ PINK1 stabilization, Parkin recruitment, LC3–mitochondria colocalization, lysosomal turnover of mitochondrial proteins, or reporter-based mitophagy assays would determine whether repeated incomplete recovery increases quality-control engagement.^12^ ^,^ ^13^ ^,^ ^135^ ^,^ ^136^ NAD⁺/NADH recovery, mitochondrial ROS resolution, and ATP restoration should be measured at matched time points to distinguish delayed recovery from reduced basal mitochondrial activity.^18-20^ ^,^ ^131^

Measurement protocols should report recovery-specific parameters rather than endpoint values alone. For SRC, oxygen consumption profiling should include basal respiration, ATP-linked respiration, maximal respiration, proton leak, non-mitochondrial respiration, and SRC at baseline and after recovery.^18-20^ For ΔΨm recovery, live-cell kinetic imaging after reversible depolarization should report the time to repolarization, the percentage of baseline ΔΨm restored, and the stability of ΔΨm after recovery.^12^ ^,^ ^104^ For redox recovery, NAD⁺/NADH restoration should be measured using targeted metabolomics, biochemical assays, or NADH autofluorescence-based approaches at matched recovery time points.^18-20^ For mitophagy, flux-based readouts should be prioritized over static marker expression, including PINK1/Parkin recruitment, LC3 colocalization with mitochondria, lysosomal inhibition-based mitochondrial protein turnover, or reporter-based mitophagy assays where available.^12^ ^,^ ^13^ ^,^ ^135^ ^,^ ^136^

Future studies should also integrate mitochondrial phenotyping with multi-omics profiling. Microbiome profiling can include 16S rRNA gene sequencing or shotgun metagenomics, while metabolite profiling should include targeted measurement of SCFAs, tryptophan-derived indoles, bile acid species, NAD⁺/NADH-related metabolites, and inflammatory lipid mediators where appropriate.^8^ ^,^ ^9^ ^,^ ^14-16^ ^,^ ^26-28^ Host-side profiling can include transcriptomics or proteomics of mitochondrial pathways, oxidative stress responses, inflammatory signaling, and mitophagy-related genes.^12^ ^,^ ^13^ ^,^ ^111^ ^,^ ^135^ ^,^ ^136^ These omics layers should be integrated with functional mitochondrial readouts, including SRC rebound, ΔΨm recovery kinetics, redox restoration, mitochondrial ROS resolution, ATP-linked respiration recovery, and mitophagy flux. Analytically, longitudinal models can determine whether microbiome-derived metabolites predict mitochondrial recovery kinetics over time. Mediation analysis can test whether microbial metabolites or immune markers mediate the relationship between microbiome composition and mitochondrial recovery outcomes. Network-based or pathway-level integration can further identify metabolite–immune–mitochondria modules associated with recovery failure or preserved mitochondrial resilience. This approach would move the field beyond cross-sectional microbiome–phenotype associations and provide a practical strategy for testing causal links between microbiome-derived signals and mitochondrial recovery capacity.

In summary, we propose that the gut microbiome influences host physiology not by dictating fixed mitochondrial states, but by modulating the probability that mitochondrial recovery succeeds or fails over time. By shifting metabolic, redox, and immune conditions, microbiome-derived signals can gradually reshape mitochondrial populations, reduce functional reserve, and increase vulnerability to stress. This persistence-driven framework positions mitochondrial recovery as a central integrator of host–microbiome interactions and provides a testable explanation for the delayed, systemic, and context-dependent nature of microbiome-associated disease. Future studies that combine time-resolved mitochondrial recovery assays with microbiome, metabolite, immune, and host-omics profiling will be essential to validate and refine this model.

**Table:** 

Study phase	Purpose	Recommended mitochondrial readouts	Recommended microbiome/host readouts	Interpretation	Representative references
Baseline profiling	Define basal mitochondrial and microbiome state	Basal respiration, ATP-linked respiration, SRC, ΔΨm, NAD⁺/NADH, mitochondrial ROS, basal mitophagy markers	16S or shotgun metagenomics, SCFAs, indoles, bile acids, cytokines, host metabolic markers	Separates basal mitochondrial activity from recovery capacity	Halfvarson et al. [8]; Lloyd-Price et al. [9]; Brand and Nicholls [18]; Dranka et al. [19]; Schmidt et al. [20]
Defined perturbation	Apply controlled metabolic, inflammatory, or oxidative stress	Immediate ΔΨm change, OCR change, ROS increase, ATP-linked respiration change	Cytokine response, metabolite shifts, immune activation markers	Defines stress sensitivity	Narendra et al. [12]; Brand and Nicholls [18]; Dranka et al. [19]; Scaduto and Grotyohann [104]; West et al. [111]
Recovery monitoring	Measure return toward baseline after stress removal	ΔΨm repolarization rate, NAD⁺/NADH restoration, SRC rebound, ATP-linked respiration recovery, ROS resolution	Metabolite persistence, immune resolution markers, host transcript/protein response	Quantifies recovery capacity and recovery failure	Brand and Nicholls [18]; Dranka et al. [19]; Schmidt et al. [20]; Scaduto and Grotyohann [104]; Murphy et al. [131]
Repeated challenge	Test whether recovery is maintained across cycles	Stability of ΔΨm recovery, SRC rebound after repeated stress, cumulative ROS, mitophagy flux	Longitudinal microbiome/metabolite changes, immune persistence markers	Defines mitochondrial resilience or cumulative recovery failure	Picard et al. [100]; Matsuda et al. [135]; Lazarou et al. [136]; Picard et al. [130]
Multi-omics integration	Link microbial signals to mitochondrial recovery	Integrated recovery score or kinetic mitochondrial parameters	Metagenomics, targeted metabolomics, transcriptomics/proteomics, immune profiling	Identifies microbial–metabolic–immune predictors of recovery dynamics	Halfvarson et al. [8]; Lloyd-Price et al. [9]; Brand and Nicholls [18]; Dranka et al. [19]; Schmidt et al. [20]
